# Accounting for long-term manifestations of *Cryptosporidium* spp infection in burden of disease and cost-of-illness estimations, the Netherlands (2013–2017)

**DOI:** 10.1371/journal.pone.0213752

**Published:** 2019-03-12

**Authors:** Susana Monge, Roan Pijnacker, Wilfrid van Pelt, Eelco Franz, Laetitia M. Kortbeek, Marie-Josée J. Mangen

**Affiliations:** 1 Centre for Infectious Disease Control Netherlands (CIb), National Institute for Public Health and the Environment (RIVM), Bilthoven, the Netherlands; 2 European Programme for Intervention Epidemiology Training (EPIET), European Centre for Disease Prevention and Control, (ECDC), Stockholm, Sweden; Sciensano, BELGIUM

## Abstract

**Background:**

Burden of disease (BoD) estimations are increasingly used to prioritize public health interventions. Previous *Cryptosporidium* BoD models accounted only for acute episodes, while there is increasing evidence of long-term manifestations. Our objective was to update *Cryptosporidium* BoD and cost-of-illness (COI) models and to estimate BoD and COI for the Netherlands in years 2013–2017.

**Methods:**

We performed a scoping literature review and drew an outcome tree including long-term manifestations for which sufficient evidence was found, such as recurrent diarrhea and joint pain. We chose the Disability-Adjusted Life Year (DALY) metric to synthesize years of life lost due mortality (YLLs) and years lived with disability due to non-fatal outcomes (YLDs). For the costs, we adopted a societal perspective accounting for direct healthcare costs, patient costs and productivity losses. Uncertainty was managed using Latin Hypercube sampling (30,000 iterations).

**Results:**

In the Netherlands in 2017, we estimated 50,000 *Cryptosporidium* cases (95% uncertainty interval (UI): 15,000–102,000), 7,000 GP visits, 300 hospitalizations and 3 deaths, resulting in 137 DALYs (95%UI: 54–255) and €19.2 million COI (95%UI: €7.2 million– €36.2 million). Estimates were highest for 2016 (218 DALYs and €31.1 million in COI), and lowest in 2013 (100 DALYs and €13.8 million in COI). Most of the BoD was attributable to YLD (≈80% of DALYs). The most important cost was productivity losses (≈90% of total COI). Long-term manifestations, including recurring diarrhea and joint pain, accounted for 9% of the total DALYs and 7% of the total COI.

**Conclusion:**

Current evidence supports the inclusion of long-term manifestations in *Cryptosporidium* models, which contribute close to 10% of the total DALYs and costs. This may be an underestimation, as we were conservative in our assumptions. *Cryptosporidium* should be considered a priority organism with respect to public health surveillance, even in industrialized countries with high hygiene standards.

## Introduction

During the last decades, there has been a growing interest in estimating the burden of disease (BoD) in order to prioritize healthcare, public health interventions and health research. Different metrics have been proposed for this, such as the Disability-Adjusted Life Year (DALY) or the Quality-Adjusted Life Year (QALY), among others [1]. The DALY metric was developed in the 1990s by the World Health Organization, the World Bank and the Harvard School of Public Health for the Global Burden of Disease project (GBD)[2], and has been widely used since [3–5]. DALYs were also adopted by the European Centre for Disease Prevention and Control (ECDC) for the Burden of Communicable Diseases in Europe (BCoDE) project in 2009, and an easy-to-use toolkit has been developed to help expand its application [6, 7]. In the Netherlands, DALYs are calculated annually for 14 food-borne pathogens [8] and for over 30 infectious diseases, including foodborne pathogens [9], to monitor their BoD and help decision making.

Many of the parameters needed for DALYs estimations are not known, or are subject to great uncertainty, requiring a number of assumptions to be made based on current knowledge. Moreover, some of these parameters are context-specific and can vary between different countries or throughout the years, depending on, for example, the quality of surveillance systems, healthcare organization and accessibility, or testing and treatment policies. As new evidence becomes available, the estimates need to be updated accordingly.

*Cryptosporidium* is a parasite that is mainly spread via the fecal-oral route from person to person or via contaminated food or water. It is more prevalent in low-resource settings, and is usually not among the health priorities for the European region [10]. Nevertheless, the infection is not so infrequent even in industrialized countries with high hygiene and sanitation standards, and it has been found to cause outbreaks, including massive outbreaks through the contamination of the public water supply [11–13]. The course of the disease can vary from asymptomatic to severe and life-threatening [14, 15]. Gastrointestinal manifestations can range from loose stool to watery diarrhea, accompanied by dehydration, weight loss, abdominal pain, nausea and/or vomiting [15]. In immunocompetent hosts, the disease is usually self-limiting, but in immunocompromised persons such as HIV infected people with CD4-counts <200/μl [14] or transplant recipients, the disease course can be more severe, even life-threatening and long lasting [15]. There is growing evidence that infection can result in long-term manifestations, including intestinal and extra-intestinal manifestations, also in immunocompetent hosts [15, 16].

Previous *Cryptosporidium* BoD estimations accounted only for the acute episode including death, but disregarding long-term manifestations [4, 10, 17, 18]. Moreover, some inputs originated from studies conducted in the 1990s, while the effective and early treatment of HIV infection nowadays [19] has greatly decreased the number of cases of opportunistic infections [20]. Finally, current diagnostic methods facilitate the detection of *Cryptosporidium*, increasing the number of diagnoses. More accurate estimations will better inform decisions around the appropriate scale of efforts directed to the surveillance, prevention and control of *Cryptosporidium*. The objective of this work is to update the models used for BoD and cost-of-illness (COI) estimation for *Cryptosporidium*, incorporating recently generated evidence, with an emphasis on long-term manifestations.

## Materials and methods

### Literature review

We performed a scoping review of published and unpublished documents (for details see supporting information in S1 File). We evaluated the quality of evidence based on external validity (mainly representativeness of the patients included and comparability with the current context in the Netherlands) and internal validity (mainly sample size, presence of a control group where appropriate and absence of other possible bias in the sample selection or data collection). We extracted data on five domains: symptoms and severity of the acute infection, including the need for medical attention and mortality; long-term manifestations and sequelae; duration of the different health outcomes; age-specific rates or age distribution of the different health outcomes; and measures of underascertainment or underreporting. Extracted data were used to draw the most plausible outcome tree and to decide on the input parameters according to best available evidence. Decisions were reviewed in a panel session. A list of articles reviewed in full-text and the information extracted is found in S1 File and summarized in Table 1.

**Table 1:** Model parameters, data sources and assumptions used in the base case and in the scenario and sensitivity analyses for the estimation of the burden of disease and costs of illness due to *Cryptosporidium*.

Model parameter	Data source	Assumptions in the base case	Scenario and sensitivity analyses
Age distribution of reported and laboratory confirmed cases	Dutch surveillance data as collected by the National Institute of Public Health and the Environment	Age distribution different for *C*. *hominis* and *C*. *parvum*	- Overall age distribution (including cases with unknown species) used for both species
Proportional share between species of reported and laboratory-confirmed cases	Dutch surveillance data as collected by the National Institute of Public Health and the Environment	2013: *C*. *hominis* = 19.43% 2014: *C*. *hominis* = 12.68% 2015: *C*. *hominis* = 48.25% 2016: *C*. *hominis* = 73.68% 2017: *C*. *hominis* = 31.75% (overall in the period)	*- C*. *hominis* 31.75% for all years - *C*. *hominis* 12.68% for all years - *C*. *hominis* 48.25% for all years
Symptomatic incident cases in the community	Tam et al. 2012 [21]	Assumed to be similar for both species. Pert distribution for the MF (applied to cases in surveillance): Most likely = 27.9 Minimum = 7.1 Maximum = 107.8	- Pert distribution for the MF: Most likely = 16.4 Minimum = 4.2 Maximum = 63.4
Incident cases presenting to the GP	Tam et al. 2012 [21]	Assumed to be similar for both species. Pert distribution for the MF (applied to cases in surveillance): Most likely = 4.6 Minimum = 2.0 Maximum = 11.2	- Pert distribution for the MF: Most likely = 2.3 Minimum = 1.0 Maximum = 5.6
Hospitalization rate of reported and laboratory-confirmed cases	Nic Lochlainn et al. 2018 [22], Dietz et al. 2000 [23], Insulander et al. 2013 [24], Hunter at al 2004 [25]	Assumed to be similar for both species. Pert distribution for hospitalization rate (applied to cases in surveillance): Most likely = 6.3% Minimum = 6.3% Maximum = 15.7%	
Multiplication factors for hospitalized cases	Mead et al. 1999 [26], Insulander et al. 2013 [24], Widerstrom et al. 2011 [27]	Assumed to be similar in both species. Pert distribution for MF (applied to number of hospitalizations and deaths): Most likely = 2.6 Minimum = 2 Maximum = 3.2	
Mortality rate of reported and laboratory-confirmed cases	Widerstrom et al. 2017 [27], Mead et al. 1999 [26]	Assumed to be similar in both species. Pert distribution: Most likely = 0% Minimum = 0% Maximum = 0.5%	Mortality rate = 0%
Age distribution of deceased cases	Cassini et al. 2018 [4], CBS: Statistics Netherlands [28]	Age distribution of deaths assumed as in CBS data [28]	Age distribution of deaths assumed as in Cassini et al. [4]
Multiplication factors for deaths	Mead et al. 1999 [26]	Assumed to be similar than MF for hospitalized cases.	
Age variation of MF (for cases in surveillance to estimate cases in the community or GP, or MF to account for underreporting of hospitalization)	De Wit et al. 2001 [29]	Varying MF by age: 0 years = 0.125 times the MF 1–4 years = 0.25 times the MF 5–14 years = 0.333 times the MF 15–64 = reference > = 65 years = 0.25 times the MF	-MF are constant across age-groups
Duration of acute episode of diarrhea in mild cases	Widerstrom 2014 [13], Corso et al. 2003 [20], Ethelberg et al. 2009 [30]	Pert distribution: Most likely: 6.5 days Minimum: 4.7 days Maximum: 9 days	
Duration of acute episode of diarrhea in moderate cases	Nic Lochlainn et al. 2018 [22], Hunter at al 2004 [25]	Uniform distribution: Minimum: 12.7 days Maximum: 16.7 days	
Duration of acute episode of diarrhea in severe patients	Widerstrom et al. 2011 [27], The ANOFEL Cryptosporidium National Network [31]	Point estimate = 20.4	
Incidence of recurrent diarrhea when the acute episode was moderate or severe	Igloi et al. 2018 [32], Hunter at al 2004 [33], Insulander et al. 2013 [24], Rehn et al. 2015 [34]	Pert distribution for incidence: Most likely = 15% Minimum = 9.1% Maximum = 19.1%	- Rates 6.7% higher in children. - Extreme scenario using the rates of overall gastrointestinal recurrent symptoms reported by [33]: 27.5%
Incidence of recurrent diarrhea when the acute episode was mild	MacKenzie et al. 1995 [12]	Incidence rate in mild cases 46% lower as compared to incidence in moderate and severe cases	-Similar incidence by severity
Number of recurrent episodes in those with recurrent diarrhea	Widerstrom 2014 [13], Adler et al. 2017 [35]	Number of episodes: 1.4 in adults, 1.5 in children	Double that number of episodes
Duration of a recurrent episode	MacKenzie et al. 1995 [12]	Pert distribution: Most likely = 2 Minimum = 1 Maximum = 15	Double that duration
Incidence of joint pain	Igloi et al. 2018 [32], Hunter et al. 2004 [33], Rehn et al. 2015 [34], Hannu et al. 2002 [36]	Pert distribution: Most likely = 6.5% Minimum = 3% Maximum = 10.7%	*-C*. *parvum* does not cause joint pain - Mild cases do not result in joint pain - Children do not develop joint pain
Severity of the joint pain	Hunter at al. 2004 [33]	Mild = 39% Moderate = 53% Severe = 8%	
Localization of the joint pain	Igloi et al. 2018 [32], Hunter at al. 2004 [33]	Location as mean of the two studies: Lower limbs (43%), Upper limbs (36%), Back (14%), Neck (9%)	
Duration of the joint pain	Hunter et al. 2004 [33], Hannu et al. 2002 [36], Locht et al. [37]	Point estimate = 10.7 days	- Point estimate = 60 days
Incidence of dizziness	Hunter at al. 2004 [33], Igloi et al. 2018 [32]	No dizziness as long-term manifestation	Uniform distribution: Minimum: 2.5% Maximum: 7.1%
Incidence of fatigue	Hunter at al. 2004 [33], Igloi et al. 2018 [32], Rehn et al. 2015 [34]	No fatigue as long-term manifestation	Uniform distribution: Minimum: 11.8% Maximum: 16.6%

Abbreviation used: MF = multiplication factors; CBS = Centraal Bureau voor de Statistiek (Statistics Netherlands); GP = General Practitioner

### Outcome trees and incident cases

**Fig 1:**
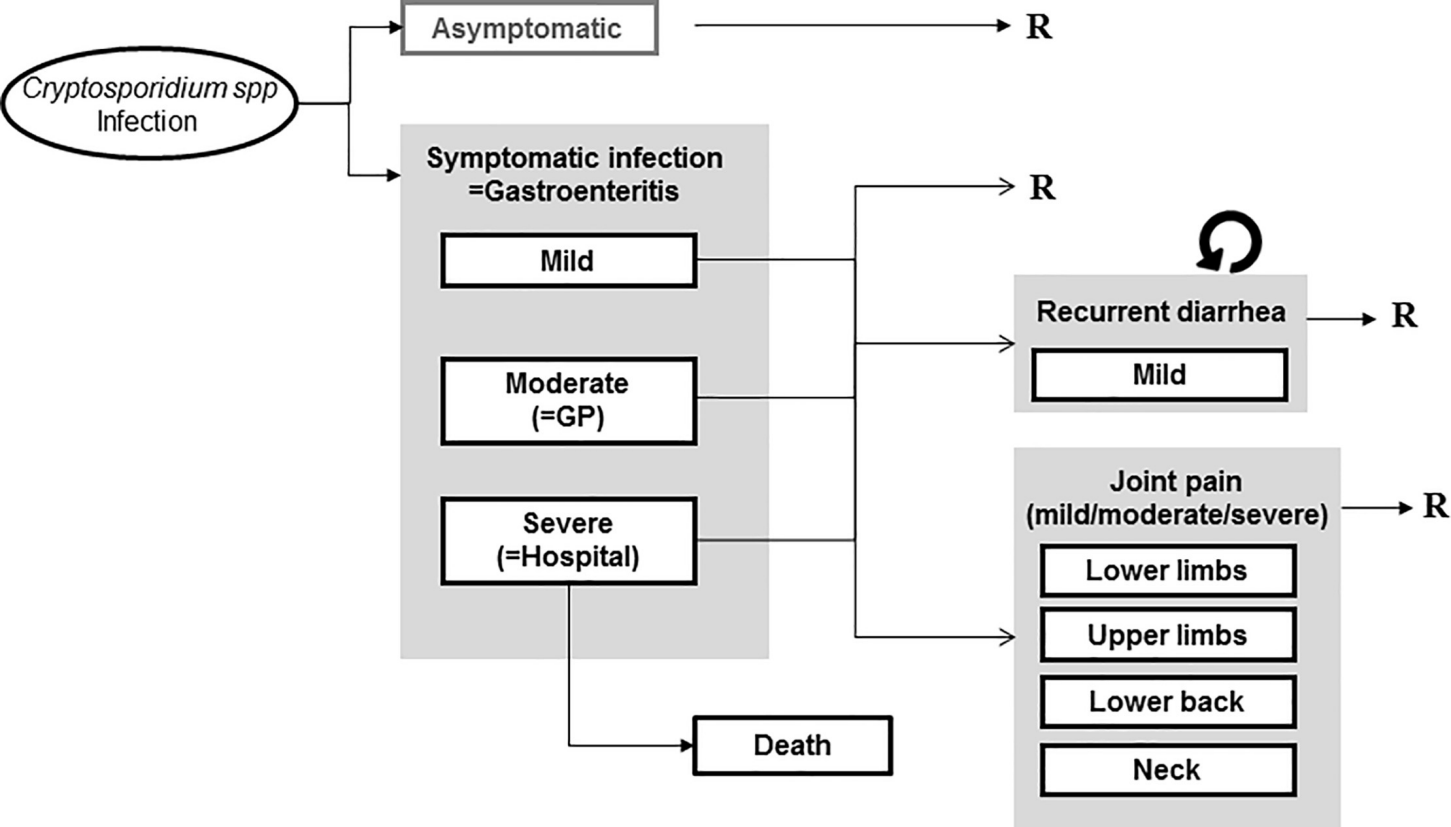
Outcome tree depicting all the possible outcomes following infection by *Cryptosporidium* spp. R: Recovery from health outcome; GP: General Practitioner.

We estimated the BoD and costs separately for the two most frequent species: *C*. *hominis* and *C*. *parvum*, since their public health implications may be different [15, 24, 33]. Evidence regarding other species is scarce, so for simplicity we assumed all *Cryptosporidium* spp. would be either *C*. *hominis* or *C*. *parvum*. As the base-case, a similar outcome tree was considered for both species (Fig 1). Since there was no clear evidence that outcomes, their duration, severity or costs differed by sex, analysis were not stratified by gender. We did consider age-specific incidence, as there were differences in health outcomes and demand of resources by age [29, 32, 35]. Finally, for simplicity reasons we decided not to account for the differences in immunocompromised patients, assuming that the population averages for duration and severity would adequately reflect the presence of immunocompromised individuals and their outcomes. Table 1 summarizes all the input data used to quantify the probabilities of transition from one outcome to the next, as well as the duration of each outcome. For more details on the rationale behind the input data for the model see S1 File. We assumed no BoD or costs of asymptomatic infections.

Acute symptomatic infections were considered mild when they did not require any medical help, moderate when they motivated General Practitioner (GP) consultation, and severe when they required hospitalization. Both the number of symptomatic *Cryptosporidium* infections in the population and the number of cases consulting a GP were estimated based on the cases captured by surveillance. For this purpose, we used the multiplication factors (MF) reported in the second study of infectious intestinal disease (IID2) from the UK [21]. This is a prospective study carried out between 2008 and 2009 that represents a sound contemporary estimation of underdiagnosis and underreporting of different gastrointestinal pathogens. However, because in the Netherlands consultation rates are classically lower [29], *Cryptosporidium* is not notifiable, and IID2 study excluded imported infections (which are around 15% of all cases in Dutch surveillance), we considered higher MF than the ones from IID2. Studies from the 90’s found that people with gastrointestinal symptoms were 3.4 times less likely to consult a GP in the Netherlands compared to the UK [29]. Therefore, we considered the MF 3.4 times higher for community cases and were more conservative (MFs twice higher) for cases in the GP. We considered lower MF in children, who have higher consultation rates in the Netherlands [29]. Hospitalization rate of lab-confirmed cases was extracted from the literature [22–25], with the most likely value (6.3%) taken from a Dutch study [22], and corrected for underreporting [24, 26, 27]. We assumed that all hospitalized cases had visited a GP and all deaths occurred among severe cases. The mortality rate and the age-distribution of deaths were subject to great uncertainty [26, 27]. In the base-case we took the age-distribution of deaths caused by “Diarrhea and gastro-enteritis of presumed infectious origin” (ICD-10 code A09) from Statistics Netherlands (2014–2017)[28]. As standard life expectancy we used the estimates from the GBD 2010 for the base-case [3].

Regarding long-term manifestations, there was evidence of recurrent gastrointestinal symptoms for both *Cryptosporidium* species up to 1 year after the acute infection [32–34, 38]. We considered only recurrent diarrhea, and no other gastrointestinal symptoms. Incidence was considered lower in mild cases [12] as compared to severe and moderate cases [24, 32–34], and higher in children <15 years [13, 35]. The number of recurrent episodes was also higher in children [13, 35]. The mean reported duration of these episodes was only 2 days [12] and therefore we assumed that they would all be mild. Joint pain was found to follow infection by *C*. *hominis* [20, 21, 32], but evidence was weaker in case of *C*. *parvum* [21]. Also, there was no evidence regarding the association of joint pain with severity of the acute episode. Therefore, in a scenario analyses we removed this outcome for mild cases and, in a different scenario we removed it for *C*. *parvum*.

Several studies have reported on dizziness [32, 33, 38] and fatigue [32–34, 38], but it is unclear if they fulfilled diagnostic criteria for specific entities (such as the chronic fatigue syndrome), or what was their severity, making it difficult to assign them a probability and a disability weight. Therefore they were not included in the base-case. Other long-term manifestations such as recurrent headaches [33], eye pain [33, 38], or irritable bowel syndrome [38] were not consistently reported.

### Disability-Adjusted Life Years

We considered all possible outcomes of *Cryptosporidium* infection (pathogen-based approach) and assigned their effects to the infection, even if some of the outcomes occur much later in time (incidence-based approach). We calculate DALYs combining YLLs and YLDs:

DALY=YLL+YLD

YLD were computed for every possible outcome *l* according to the following formula:

$$YLD=\sum n_{l}^{a}*t_{l}^{a}*{dw}_{l}$$

Where $n_{l}^{a}$ is the number of cases from a given age-group with outcome *l*, $t_{l}^{a}$ is the duration of that outcome for that particular age-group, and *dw*_*l*_ is the disability weight associated to outcome *l*. Disability weights were taken from Haagsma et al. [39] and were assumed to be homogeneous across age-groups. Table 2 shows the disability weights that were applied to every different outcome in the model. We did not take into consideration that the presence of multiple simultaneous outcomes could result in a multiplicative effect over the disability weight and assumed an additive model, where the disability for each outcome is independent on other coexisting outcomes.

**Table 2:** Disability weights extracted from Haagsma et al. [39] for each of the health outcomes of the *Cryptosporidium* burden of disease model.

Outcome in *Cryptosporidium* model	Health state from Haagsma et al.	Mean disability weight
Symptomatic infection (mild)	Diarrhea, mild	0.073 (0.061–0.092)
Symptomatic infection (moderate)	Diarrhea, moderate	0.149 (0.12–0.182)
Symptomatic infection (severe)	Diarrhea, severe	0.239 (0.202–0.285)
Recurrent diarrhea (mild)	Diarrhea, mild	0.073 (0.061–0.092)
Joint pain in lower limbs (mild)	Musculoskeletal problems, lower limbs, mild	0.027 (0.021–0.032)
Joint pain in lower limbs (moderate)	Musculoskeletal problems, lower limbs, moderate	0.094 (0.08–0.12)
Joint pain in lower limbs (severe)	Musculoskeletal problems, lower limbs, severe	0.134 (0.11–0.165)
Joint pain in upper limbs (mild)	Musculoskeletal problems, upper limbs, mild	0.041 (0.032–0.05)
Joint pain in upper limbs (moderate)	Musculoskeletal problems, upper limbs, moderate	0.138 (0.114–0.167)
Joint pain in upper limbs (severe)	*Assumed similar to joint pain in upper limbs (moderate)*	-
Joint pain back (mild)	Low back pain, mild	0.024 (0.018–0.03)
Joint pain back (moderate)	Low back pain, moderate	0.060 (0.05–0.074)
Joint pain back (severe)	*Assumed similar to joint pain back (moderate)*	-
Joint pain neck (mild)	Neck pain, acute, mild	0.062 (0.05–0.075)
Joint pain neck (moderate)	*Assumed similar to joint pain back (mild)*	-
Joint pain neck (severe)	Neck pain, acute, severe	0.224 (0.19–0.268)
Dizziness (severity not specified)	Infectious disease, acute episode, mild	0.007 (0.005–0.01)
Fatigue (not specified if compatible with chronic fatigue syndrome)	Infectious disease, acute episode, mild	0.007 (0.005–0.01)

YLLs were given by the remaining life expectancy at age of death (*e*^*a*^), accumulated for all deaths due to the infection in each age group (*n*^*a*^), following the formula:

$$YLL=\sum n^{a}*e^{a}$$

In this case, only deaths from acute infections were considered, since none of the long-term manifestations have been reported to result in death.

DALYs were discounted at 1.5%, according to the Dutch guidelines for health economic evaluation [40].

### Costs

We adopted a societal perspective, taking into account costs from the healthcare sector, from the patient himself or his/her family and other sectors. Direct healthcare costs (DHC) included costs for medical consultations, hospital stay, ambulance transport, diagnostic tests, prescribed medication, and temporary stays in nursing homes. Patient costs (PC) included cost for transport to consultations, over-the-counter medication (OCM) and extra-diapers for babies. Other sector costs (OSC) included loss of productivity (paid and unpaid work, where available) for either the sick person or a caregiver, for non-fatal cases. For fatal cases, loss of productivity was computed taking the friction time approach, i.e. the mean time needed to find a replacement worker [40, 41]. The detailed assumptions made for computing the costs can be found in S1 File. Costs were not discounted as we did not consider any costs occurring beyond one year after the infection.

Assumptions regarding consultation habits, frequency of diagnostic fecal tests, prescribed medication and over the counter medication (OCM), transportation and hours of productivity lost during an acute gastroenteritis episode were taken from Mangen et al. [18] and are also summarized in S1 File. Unitary costs were taken, when available from the Dutch healthcare institute [40], and otherwise from Mangen et al. [18]. For the mean duration of hospital stay we re-analyzed the data from a Dutch case-control study [22] by age group: 5.3 days for <5 years of age; 3.6 days for 5–14 years of age; 5.7 days for 15–64 years of age; and 7.0 days for ≥65 years of age.

As the base-case regarding recurrent diarrhea, we considered it did not motivate medical consultations since severity was assumed mild. OCM costs for a recurrent diarrhea episode were corrected for the shorter duration as compared to the acute episode. For joint pain, we considered that 100% of severe, 41.5% of moderate and 0% of mild cases will visit a GP [33] and a similar proportion would take anti-inflammatory drugs and pain-killers as OCM during half the duration of the pain. We assumed no diagnostic tests, hospital admissions or emergency department visits. Loss of productivity was zero for mild cases, and for moderate and severe cases, equivalent to a moderate episode of post-infectious reactive arthritis as reported by Mangen et al. [17].

### Model and management of uncertainty

The model was built in MS Excel using the add-in software Palisade @Risk 7.5. A probability distribution was chosen for most parameters to manage uncertainty, whereby using either a Pert-distribution, a uniform, or a discrete distribution. A value was drawn from such distributions iteratively using Latin Hypercube sampling with 30,000 iterations. We computed the mean and the 95% uncertainty interval (95% UI), corresponding to the 2.5^th^ and 97.5^th^ percentiles of the results’ distribution. Population heterogeneity in the duration of the outcomes and the disability weights were considered to be well represented by the population mean. For input parameters with model uncertainties, we ran different scenarios with varying assumptions, and varied input parameters in univariate and multivariate sensitivity analyses.

### Scenario and sensitivity analyses

Table 1 shows the assumptions and input parameters used in different scenario and sensitivity analyses. Sensitivity and scenario analyses were conducted assuming different age distributions for incidence and mortality, lower MFs, no variation of MF across age-groups, or no deaths caused by *Cryptosporidium*, among others. For recurrent diarrhea, double incidence and duration was applied as sensitivity analysis, and also different incidence by age and severity of the acute episode. For joint pain, as scenarios we did not consider it a) for *C*. *parvum*, b) for mild cases or c) for children, and d) we assumed a higher duration of a joint pain episode. Dizziness and fatigue were included in the model in a scenario analysis, assuming similar duration as for joint pain in absence of reliable data.

## Results

In the years between 2013 and 2017 (Table 3), *Cryptosporidium* caused between 100 and 218 DALYs per year, with a higher burden estimated in the years 2015 and 2016, in which a higher number of cases was reported to the surveillance system. The increase was mainly due to an increase in *C*. *hominis* (160 DALYs in 2016 vs. 12 in 2014), while the BoD of *C*. *parvum* was more stable at between 58–96 DALYs (Table 3). In the same period, the associated annual costs were between 13.8 million € (M€) and 31.1 M€ (Table 3). In the year 2017, *Cryptosporidium* caused a total of 137 DALYs and 19.2 M€ in COI in the Netherlands (i.e. 8 DALYs and 1.1 M€ per million inhabitants).

**Table 3:** Average estimates and 95% Uncertainty Interval between brackets for the number of cases with different health outcomes, Disability Adjusted Life Years (DALYs) and costs of illness (COI) of *Cryptosporidium* infection in the Netherlands for the years 2013–2017 using the base-case model.

		2013	2014	2015	2016	2017
**Overall**						
Surveillance data	Number of reported cases/year (nationally)	973	988	1769	2108	1332
Estimated number of cases/y ear	Total symptomatic (x 1,000)	37 (11–74)	37 (11–75)	67 (20–135)	80 (24–161)	50 (15–102)
	GP consultations (x 1,000)	5 (2–8)	5 (2–8)	9 (4–15)	11 (5–18)	7 (3–11)
	Hospitalizations (x 1,000)	0.2 (0.1–0.3)	0.2 (0.1–0.3)	0.4 (0.3–0.5)	0.4 (0.3–0.6)	0.3 (0.2–0.4)
	Case fatalities	2 (0–7)	2 (0–7)	4 (0–12)	5 (0–14)	3 (0–9)
	Cases of joint pain (x 1,000)	2 (1–5)	2 (1–6)	4 (1–10)	5 (1–12)	3 (1–7)
	Cases with recurrent diarrhea (x 1,000)	3 (1–7)	3 (1–7)	6 (2–12)	7 (2–15)	4 (1–9)
DALYs/year and costs/year	Total DALYs* –discounted 1.5%	100 (39–187)	102 (39–191)	183 (72–337)	218 (86–401)	137 (54–255)
	Total DALYs–undiscounted	103 (39–196)	104 (40–198)	187 (73–349)	223 (87–414)	140 (54–264)
	DALYs* per million inhabitants	6 (2–11)	6 (2–11)	11 (4–20)	13 (5–24)	8 (3–15)
	Total costs^#^ (million €)	13.8 (5.1–26.1)	14.0 (5.2–26.6)	25.9 (9.9–48.6)	31.1 (12.0–58.3)	19.2 (7.2–36.2)
	Costs^#^ per million inhabitants (million €)	0.8 (0.3–1.6)	0.8 (0.3–1.6)	1.5 (0.6–2.5)	1.8 (0.7–3.4)	1.1 (0.4–2.1)
**By species**						
*C*. *hominis*	Total symptomatic/year (x 1,000)	7 (2–14)	5 (1–9)	32 (10–65)	59 (17–119)	16 (5–32)
	Total DALYs*/year	19 (6–39)	12 (4–28)	87 (32–163)	160 (63–295)	44 (16–84)
	Total Costs^#^/year (million €)	2.6 (0.9–5.0)	1.7 (0.6–3.3)	11.8 (4.3–22.5)	21.3 (7.8–40.9)	5.8 (2.1–11.2)
*C*. *parvum*	Total symptomatic/year (x 1,000)	30 (9–60)	33 (10–66)	35 (10–70)	21 (6–43)	34 (10–69)
	Total DALYs*/year	81 (32–152)	89 (35–166)	96 (38–178)	58 (22–109)	94 (37–174)
	Total Costs^#^/year (million €)	11.0 (4.1–21.0)	12.0 (4.4–22.9)	14.3 (5.8–26.5)	9.7 (4.1–17.4)	13.3 (5.1–25.0)

*The DALYs are discounted at 1.5% per year according to the Dutch guidelines for health economics(40)

^#^costs are not discounted since all costs happened within the year of infection.

The biggest share of the disease burden was due to YLD, representing 83% of the total DALYs in year 2017 (114 YLD and 23 YLL, Table 4). The majority of costs were due to productivity losses, which accounted for 17.6 M€ in that same year, 91.7% of the total COI (Table 4). The cost per case was slightly higher for *C*. *parvum*. For example in year 2017, costs were €404 for *C*. *parvum* and €377 for *C*. *hominis*.

**Table 4:** Average estimates and 95% Uncertainty Interval between brackets for Disability Adjusted Life Years (DALYs) and costs of illness (COI) of *Cryptosporidium* infection in the Netherlands: base-case model for year 2017.

		DALYs*				Costs^#^				
		YLD /year	YLL* /year	Total DALYs* /year	DALYs* per 1,000 cases	DHC/year (million €)	Patient costs/year (million €)	Productivity losses/year (million €)	Total costs/year (million €)	Costs per 1,000 cases (*1,000 €)
**Overall**		114 (43–216)	23 (0–83)	137 (54–255)	3 (2–5)	1.4 (0.9–2)	0.2 (0.1–0.4)	17.6 (6.1–34)	19.2 (7.2–36)	396 (341–506)
**Per million inhabitants**		7 (3–13)	1 (0–5)	8 (3–15)	-	0.08 (0.05–0.12)	0.01 (0.01–0.02)	1 (0.4–2)	1.1 (0.4–2.1)	-
**Acute episode**		101 (39–191)	23 (0–83)	125 (49–231)	2.7 (1.7–5.1)	1.3 (0.9–1.9)	0.2 (0.1–0.4)	16 (5.7–31)	17.6 (6.7–33)	364 (316–469)
**Long-term manifestations**	**Diarrhea**	5 (1–15)	0 (0–0)	5 (1–15)	0.1 (0.03–0.24)	0 (0–0)	0.02 (0.01–0.03)	1.02 (0.17–3.1)	1 (0.2–3.2)	21 (6–50)
	**Joint pain**	8 (2–17)	0 (0–0)	8 (2–17)	0.15 (0.09–0.22)	0.03 (0.01–0.08)	0.01 (0–0.01)	0.5 (0.13–1.12)	0.5 (0.1–1.2)	11 (6–15)
	**Total**	13 (3–29)	0 (0–0)	13 (3–29)	0.3 (0.1–0.4)	0.03 (0.01–0.08)	0.02 (0.01–0.04)	1.52 (0.36–3.96)	1.6 (0.4–4.1)	31.6 (15.4–61.4)
**By species**	***C*. *hominis***	36 (14–69)	7 (0–33)	44 (16–84)	2.9 (1.9–5.8)	0.4 (0.3–0.6)	0.07 (0.03–0.13)	5.3 (1.8–10.5)	5.8 (2.1–11.1)	377 (332–463)
	***C*. *parvum***	78 (30–148)	16 (0–60)	94 (37–174)	2.9 (2–5.3)	0.9 (0.6–1.3)	0.15 (0.05–0.28)	12.2 (4.4–23.5)	13.3 (5.1–25)	404 (344–526)

*The DALYs are discounted at 1.5% per year according to the Dutch guidelines for health economics(40)

^#^costs are not discounted since all costs happened within the year of infection.

Long-term manifestations accounted for 13 DALYs in year 2017 (9% of the total 137 DALYs), of which 8 DALYs were caused by joint pain and 5 DALYs by recurrent diarrhea (Table 4). They produced a cost of 1.6 M€ (8% of the total 19.2 M€ of COI), of which 1.0 M€ was attributable to recurrent diarrhea and 0.5 M€ to joint pain (Table 4). In previous years, i.e. pre-2017, the total number of DALYs and costs caused by long-term manifestations were different, but the proportion they represented with regards to total DALYs and costs was similar; for instance in 2016, of a total of 218 DALYs and 31 M€ of COI, 20 DALYs (9%) and 2.5 M€ (8%), respectively, resulted from long-term manifestations (S1 File).

Productivity losses accounted for 1.5 M€ (93.8%) of the 1.6 M€ caused by long-term manifestations, in year 2017.

### Scenario and sensitivity analyses

**Fig 2:**
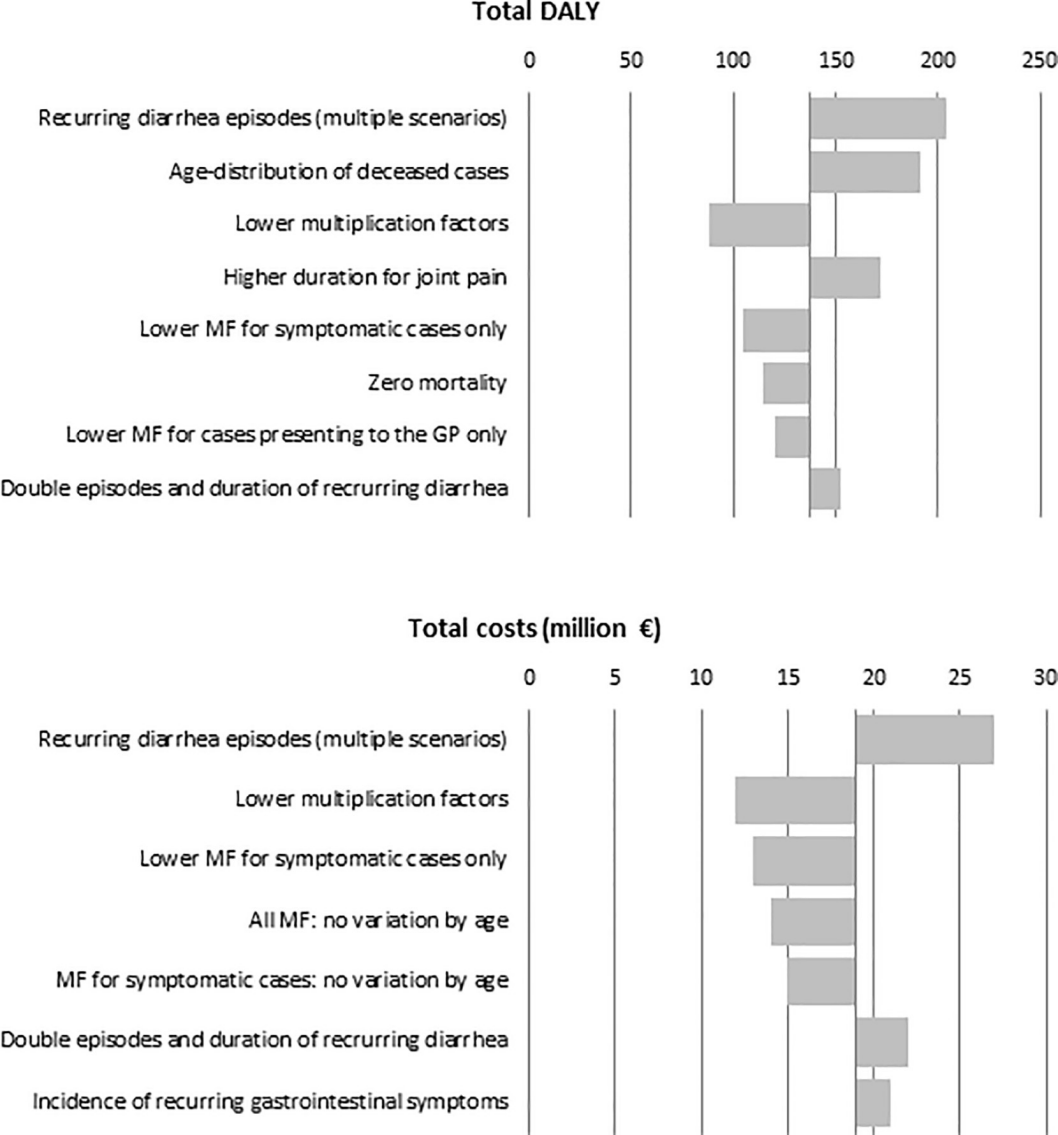
Average Disability Adjusted Life Years (DALYs) and average Cost of Illness (COI) estimated under different scenario and sensitivity analyses in the Netherlands in 2017. Note: Only those that produced a change greater than 10% in the estimation when compared to the baseline model are shown. For full details see S1 File.

The impact of key assumptions was explored in 28 different scenario and sensitivity analyses applied to estimations of the year 2017 (detailed results in S1 File). Analyses producing a change greater than 10% of the total DALYs or total costs are shown in Fig 2. The greatest change was observed when applying multiple scenarios of recurrent diarrhea simultaneously: incidence of recurrent diarrhea in mild *Cryptosporidium* cases similar to moderate and severe, with no differences between age groups, with similar severity as acute episodes and with twice the number of recurrent diarrhea episodes per patient and a two-fold duration of these episodes. This increased total DALYs per year from 137 to 204 (95%UI: 74–428) and total costs per year from 19 to 27 M€ (95%UI: 9–54). Using the age distribution of deceased cases from European surveillance data [4] increased the proportion of cases in younger ages and increased the estimated DALYs to 191 (95%UI: 62–399) versus 137 in the base case. Using lower multiplication factors to derive the number of symptomatic cases and cases in the GP from surveillance reports decreased DALYs down to 88 (95%UI: 33–172) and total costs down to 12 M€ (95%UI: 5–22).

When we applied all scenarios that resulted in lower DALYs and/or lower COI simultaneously, DALYs was 61 (95%UI: 23–115) and COI 8 M€ (95%UI: 3–15) per year. Oppositely, when applying all more liberal scenarios simultaneously, these figures went up to 395 DALYs (95%UI: 121–846) and 34 M€ (11–74) per year.

## Discussion

This is the first estimation of burden of disease and cost of illness of *Cryptosporidium* infection to account for long-term manifestations. Recurrent diarrhea and joint pain together accounted for 9% of the total DALYs and 8% of the total COI. Overall, *Cryptosporidium* produced 137 DALYs and a cost of 19.2 M€ in the Netherlands in 2017, although this varied by year. The highest burden is observed in years 2015 and 2016, where there was an increase in sporadic cases reported to surveillance, mainly of *C*. *hominis*, resulting in 218 DALYs and 31.1 M€ in COI in 2016; no single source or prominent risk factor was found to explain this increase [22]. This protozoon should be among the priorities for public health surveillance in the Netherlands.

Long-term manifestations following acute cryptosporidiosis have been consistently reported in the literature and future models should consider these within the outcomes associated to infection. The proportion of the total burden of disease due to long-term manifestations was modest compared to what has been observed for other gastrointestinal diseases. For example, the inclusion of irritable bowel syndrome (IBS) as a sequelae increased the BoD by 86% for *Salmonella*, 92% for *Campylobacter*, and 151% for *Shigella* [42]. In more recent studies, sequelae as a whole, including also reactive arthritis, inflammatory bowel disease and, for *Campylobacter*, Guillain-Barré syndrome, accounted for around 70% of the total BoD for *Campylobacter*, and 60% for *Salmonella* [8, 10].

The impact of recurrent diarrhea episodes in *Cryptosporidium* was much lower than IBS for other pathogens due to the much shorter duration and severity we accounted for. Given the mostly conservative assumptions, our results are probably an underestimation. More liberal scenarios estimated a higher burden, but were still far from the impact associated to IBS. Likewise, the lack of more detailed information on severity of the joint pain and its compatibility with a diagnosis of reactive arthritis, together with the existence of only one study addressing its duration, have probably resulted in an underestimation of its impact. Finally, because we included only sequelae with high evidence and sufficient data allowing us to parameterize the model, dizziness and fatigue were only included in a sensitivity analysis and with very conservative assumptions. Chronic fatigue syndrome has been described following other protozoan infections such as giardiasis [43], and could therefore also explain part of the fatigue cases following cryptosporidiosis. Future research could try to better characterize if such manifestations could correspond to established clinical entities, and better define their severity and duration, as well as whether their probability depends on the severity of the acute infection.

It is difficult to compare our estimates with previous studies due to the multiple updates in the input parameters used for the model. The report on disease burden of food-related pathogens in the Netherlands for year 2017 [8] estimated a higher number of cases, namely 69,000, compared to 50,000 in our model, a lower number of cases visiting the GP (4,200 vs.7,000), a higher number of hospitalizations (600 vs. 300) and more deaths (4 vs.3), although 95% uncertainty intervals overlapped in all cases. This resulted in 120 DALYs in 2017 (undiscounted), very similar to the 125 DALYs in our study if we accounted only for acute infections. The estimated costs in 2017 were 17 M€ vs. 18 M€ in our study if accounting only for the acute episodes.

The updated estimates accounting for long-term manifestations, of 137 DALYs for year 2017, would still have placed Cryptosporidium in the 13^th^ place of the 14 food-related pathogens evaluated in the Netherlands for that year, only above *Bacillus cereus* and close to the 150 DALYs estimated for Shiga toxin-producing *Escherichia coli* (STEC) O157 [8], although this ranking varies year to year. When establishing comparisons, however, we need to consider that previous estimations have accounted for sequelae of *Campylobacter*, *Salmonella*, STEC O157, *Listeria monocytogenes* and *Toxoplasma gondii* but not for any of the bacterial toxins, viral or protozoan pathogens, whose burden may be currently underestimated [10, 18]. In a recently published study, Cassini et al. [4] estimated the burden of selected infectious diseases in the European Union and European Economic Area countries in the period 2009–2013. They estimated 0.8 DALYs per million inhabitants due to *Cryptosporidium*, higher than our 0.6 estimated for 2013, more so taking into account that they did not consider long-term manifestations and the assumed incidence in our study was higher. This could be mostly explained by the different age distribution of fatal cases, which in European surveillance data occur at younger ages as compared to what is seen in the Netherlands, although differences in other assumptions could also play a role. In the ranking by Cassini et al. [4], *Cryptosporidium* had the lowest burden among the foodborne pathogens, including *Campylobacter*, *Salmonella*, STEC, *Listeria*, hepatitis A virus, *Shigella*, congenital toxoplasmosis, and *Giardia*. Among intestinal parasites, a recent prioritization exercise using multi-criteria decision analysis ranked *Cryptosporidium* in second place for the countries in Northern Europe, after *Echinococcus multilocularis* [44].

As an overall limitation, the uncertainty in some of the assumptions was high, and although we tried to account for this by using a probabilistic approach, still our results may be biased by the particular characteristics of the studies used to derive our input parameters. The uncertainty was also reflected in the wide variation when applying all scenarios simultaneously, which resulted in a number of DALYs as low as 61 or as high as 395, and a COI of 8 M€ up to 34 M€ in 2017. Finally, these estimations were very specific of a particular context and extrapolation to countries with different epidemiological profiles, different structure, accessibility and quality of health systems, or different age structure or share of immunocompromised patients, needs to be done with caution. In low-resource settings with high incidence in younger ages [15] the burden caused by this protozoon is likely to be several orders of magnitude higher than the one estimated in our study.

In conclusion, current evidence supports the inclusion of long-term manifestations in *Cryptosporidium* BoD models which contribute close to 10% of the total DALYs and costs. The burden and costs of *Cryptosporidium* under our model was higher than previously estimated, reinforcing the need to consider it a priority organism with respect to public health surveillance, even in an industrialized country with high hygiene standards such as the Netherlands.

## Supporting information

S1 File Supplementary material: Accounting for long-term manifestations of Cryptosporidium spp infection in burden of disease and cost-of-illness estimations, the Netherlands (2013–2017). (DOCX)
