# Supplementary material for: Accounting for long-term manifestations of Cryptosporidium spp infection in burden of disease and cost-of-illness estimations, the Netherlands (2013–2017)
Source: PLoS One. 2019 Mar 12;14(3):e0213752. doi: 10.1371/journal.pone.0213752 (PMC6413911; doi:10.1371/journal.pone.0213752)
Supplement: S1 File — (DOCX) [file pone.0213752.s001.docx]

**Supplementary material: Accounting for long-term manifestations of *Cryptosporidium spp* infection in burden of disease and cost-of-illness estimations, the Netherlands (2013-2017)**

Susana Monge, Roan Pijnacker, Wilfrid van Pelt, Eelco Franz, Laetitia M. Kortbeek, Marie-Josée J. Mangen

Contents

[Literature review 2](#_Toc525330806)

[Cryptosporidium spp burden of disease model 8](#_Toc525330807)

[Disability Weights 25](#_Toc525330808)

[Costs of Cryptosporidium spp 27](#_Toc525330809)

[Results of the sensitivity and scenario analyses 31](#_Toc525330810)

# Literature review

We performed a scoping review of published and unpublished documents using PubMed and Google without any language restrictions. The specific terms used in the searches for *Cryptosporidium spp*., as well as the number of documents reviewed are shown in **Figure S1**. We extracted data on five domains: symptoms and severity of the acute infection, including the need for medical attention and mortality; long-term manifestations and sequelae; duration of the different health states; age-specific rates or age distribution of the different health-states; and measures of underascertainment or underreporting. We evaluated the quality of the evidence based on external validity (mainly representativeness of the patients included and comparability with the current context) and internal validity (mainly sample size, presence of a control group where appropriate, absence of other possible bias in the sample selection or data collection). We extracted the data of interest and selected the best available evidence to draw the outcome tree and decide on the input data for the models. Decisions were reviewed in a panel session. **Table S1** offers a brief description of the revised bibliography and of which information was extracted.

Estimations were performed in this study for years 2013 - 2017.

**Figure S1. Methods and results of the scoping review for *Cryptosporidium spp***

**Ad-Hoc Search**

Ad hoc search using references from papers resulting from PubMed searches, as well as grey literature retrieved through Google or expert’s knowledge

**PubMed Search 2**

(outbreak[TITLE] OR outbreaks[TITLE]) AND(cryptosporidium[TITLE] OR cryptosporidiosis[TITLE])

**PubMed Search 1**

((Cryptosporidium OR Cryptosporidiosis) AND (risk OR incidence OR sequelae OR duration OR severity OR hospitalization) NOT prevention NOT water NOT weather NOT animal NOT in vitro) AND ("2007"[Date - Publication]: "2018"[Date - Publication])

**N = 408**

**N = 249**

**3 duplicates**

**Reviewed full-text**

**N = 34**

**Reviewed full-text**

**n = 20**

**Reviewed full-text**

**n = 47**

**Data extracted for model inputs**

**n = 26**

**Table S1. Summary of the literature reviewed for *Cryptosporidium spp* ordered by first author alphabetically.**

| **Short reference** | **Country** | **Year** | **Brief description** | **Summary of relevant findings for the study / model parameters extracted** |
| --- | --- | --- | --- | --- |
| Abal-Fabeiro et al 2015 (1) | Spain | 2000-2008 | Retrospective study of patients with diarrhea that underwent etiological investigation and had lab-confirmed cryptosporidiosis. | - Hospitalization rate overall. The authors report higher hospitalization rate in *C. parvum*, but due to lack of more supporting evidence, difference in hospitalization rates between species were not accounted for. |
| Adler et al. 2017 (2) | Sweden | 2010 | Same *Cryptosporidium* outbreak as in Widerstrom et al. 2014 (3), focus on children <15 years. | - Duration of acute episode of diarrhea, with no difference between age groups or by sex.  - Recurrence of diarrhea after >2 days of normal stools overall, with no difference between age groups or by sex.  - No difference in symptoms by age group. |
| Bouzid et al. 2013 (4) | England & Wales | 1989-2008 | Review paper. It contains some data on age distribution of *C. parvum* (the only species that is notifiable) in England & Wales. | - Age distribution of *C. parvum* notifications from 20 years of surveillance. |
| Cassini et al. 2018 (5) | Europe | 2013 | Burden of disease estimation study, with some input data from European surveillance (TESSy). | - Distribution by age of *Cryptosporidium* cases (with no information on the species).  - Distribution by sex, not used in this study. |
| Corso et al. 2003 (6) | USA | 1993 | Same *Cryptosporidium* outbreak as in Mac Kenzie et al. 1995 (7). Analysis of medical costs and productivity losses. | - Severity of the acute episode.  - Duration of hospitalization.  - Consultation, emergency department attendances, self-medication or drug prescription rates difficult to extrapolate to the Netherlands and not used. |
| Dietz et al. 2000 (8) | USA | 1997-1998 | Surveillance data of notified *C. parvum* infections. High proportion of HIV positive individuals: 44% of all cases, 74% of hospitalized, 50% of deceased (but 100% of deceased patients with known status). | - Hospitalization rate.  - Fatality rate was not used, due to the very high proportion of HIV infection among the deceased. |
| Ethelberg et al. 2009 (9) | Denmark | 2005 | Outbreak of *C. hominis* involving 13 cases, case-control study followed by a cohort study. | -Duration of acute episode. |
| Haagsma et al 2010 (10) | The Netherlands | 2010 | Estimation of burden of disease due to Irritable Bowel syndrome in the Netherlands. | - Proportion and duration with irritable bowel syndrome. |
| Hoxie et al. 1997 (11) | USA | 1993 | Same *Cryptosporidium* outbreak as in Mac Kenzie et al. 1995 (7). | - *Cryptosporidium* attributable morality after vs. before the outbreak; 85% of deceased patients had AIDS as a contributing cause, therefore estimates were not used. |
| Hunter et al. 2004 (12) | The UK | 2001-2002 | Case-control study of sporadic cryptosporidiosis with species determination for 191 patients, including both *C. hominis* and *C. parvum.* | - Duration of acute episode of diarrhea, with no variation by species.  - Hospitalization rate and duration of hospitalization, with no variation by species.  - Proportional share between the two species not extracted, as more representative data available.  - Age distribution by species not used, as more representative data available. |
| Hunter et al. 2004 (13) | The UK | 2001-2002 | Same *Cryptosporidium* case-control study as in (12), here reporting results of self-reported symptoms at 2 months of follow-up. | - Incidence, severity, duration and requirement of medical attention due to long-term health effects, overall and by species. |
| Igloi et al. 2018 (14) | The Netherlands | 2013-2016 | Follow-up study of the *Cryptosporidium* cases from the case-control study in Nic Lochlainn et al. 2018 (15), 4 months after the acute episode. Case-cross over design, with self-report of different symptom before, during and after the disease and comparison with controls. Because for some outcomes the controls are substantially different than the cases before the illness, only the comparison before-after was used. | - Proportion developing long-term manifestations and sequelae after the acute episode.  - Location of joint pain (proportional implication of different joints). |
| Insulander et al. 2008 (16) | Sweden | 2008 | Local outbreak of *C. parvum.* | - Hospitalization rate. |
| Insulander et al. 2013 (17) | Sweden | 2008 | Description of cases from local surveillance, linked and not linked to outbreaks, including both *C. hominis* and *C. parvum*, with no control group. | - Hospitalization rate, with no difference between species.  -Duration of acute episode of diarrhea, longer for *C. parvum* but not accounted for due to contradictory evidence.  - Incidence of intermittent diarrhea , assumed to be recurrent diarrhea. |
| Mac Kenzie et al. 1995 (7) | USA | 1993 | Massive outbreak of *C. parvum.* | - Rate of recurrence of watery diarrhea after apparent recovery (with at least 2 days of normal stools). |
| Mead et al. 1999 (18) | USA | 1995-1998 | Population-level estimation of food-related illness and death based on multiple surveillance systems and other sources. | - Hospitalization rate.  - Case-fatality rate.  - MF for underascertainment/underreporting of hospitalized and fatal cases. |
| Mc Cann et al. 2014 (19) | The UK | 2010 | Local *Cryptosporidium* outbreak recruiting 48 probable cases and 53 non-cases of *C. hominis.* | - Duration of illness.  - Hospitalization rate. |
| Nic Lochlainn et al. 2018 (15) | The Netherlands | 2013-2016 | Case-control study involving 609 cases of laboratory confirmed cryptosporidiosis, recruited by GPs, and 1548 controls throughout 3 years. 56% of them were speciated, resulting in identification of *C. hominis*, *C. parvum*, and a percentage of unknown species. | **-** Duration of acute episode of diarrhea, longer for *C. hominis*, but difference not accounted for due to contradictory evidence.  - Hospitalization rate (with no difference by species).  - Duration of hospitalization (with no difference by species). |
| Rehn et al. 2015 (20) | Sweden | 2010-2011 | Two outbreaks of *C. hominis* in two different cities in Sweden, one same outbreak as in (3). Follow-up questionnaire up to 11 months, but at different and unmeasured times after the acute disease. | - Incidence of long-term manifestations. |
| Schierenberg et al. 2017 (21) | The Netherlands | 2012 | Cohort study using routine care data of 225 GPs. Episodes of GE and tests of fecal samples were extracted from electronic records for 500 patients. | - Proportion of patients with GE visiting a GP that will get a fecal sample analyzed |
| Stiff et al. 2017 (22) | The UK | 2012 | Longitudinal study in outbreak-associated cases of *C. parvum* up to 1 year after the acute infection. Response rates of 27% and 20% at 6 and 12 months, respectively. | - Duration of the acute episode .  - Hospitalization rate.  - Duration of hospitalization . |
| Tam et al. 2012 (23) | The UK | 2008-2009 | Prospective, community cohort study and prospective study of GP presentation addressing multiple infectious intestinal diseases (including *Cryptosporidium*), with 6836 participants. Species is not specified, but is probably only *C. parvum*, since this is the only notifiable one in the UK. | - MFs to estimate number of cases in the community and number of cases visiting a GP based on cases reported to national surveillance systems. |
| The ANOFEL *Cryptosporidium* National Network 2010 (24) | France | 2006-2009 | Network of 38 hospital parasitology laboratories that voluntarily reported new cases of confirmed human cryptosporidiosis. | - Information on species other than *C. hominis* and *C. parvum*  - Share between immunocompromised and immunocompetent patients among *Cryptosporidium* cases. |
| Widerstrom et al. 2014 (3) | Sweden | 2010 | Retrospective study of a large waterborne outbreak of *C. hominis*. Included individuals with ≥3 episodes of diarrhea daily and/or watery diarrhea. | - Duration of the acute episode overall and by age.  - Recurrence of diarrhea after >2 days of normal stools overall and by sex. |
| Widerstrom et al. 2011 (25) | Sweden | 2010 | Same *Cryptosporidium* outbreak as in (3), describing in depth hospitalized cases during the outbreak. | - Hospitalization rate.  - Duration of diarrhea in hospitalized patients.  - Mortality rate. |
| De Wit et al. 2001 (26) | The Netherlands | 1996-1999 | Comparison of GE cases that consulted a GP with those who did not in a community-based study . | - Consultation rates for people with GE. |

GP: General Practitioner, GE: Gastroenteritis, MF: Multiplicationfactor

# Burden of disease model

***Outcome tree***

We estimated the BoD separately for the two most frequent *Cryptosporidium* species: *C. hominis* and *C. parvum,* since their public health implications may be different (13, 17, 27). In the base case, we drew a common outcome tree for *C. hominis* and *C. parvum* (**Figure S2**). However, there is some evidence that outcomes might differ between the two species, for example, because the evidence for the association between *C. parvum* and joint pain is scarce as compared to *C. hominis*, where it is more established (13, 14, 20). Therefore in a scenario analysis we drew a different outcome tree for *C. parvum* removing joint pain as a possible outcome.

The evidence regarding other species is so scarce, so for simplicity we assumed all *Cryptosporidium spp.* cases in the Netherlands would be either *hominis* or *parvum*. Other species are also detected among hospitalized or complicated patients (24), but are not routinely investigated in fecal samples, therefore are largely underdiagnosed. Our results could therefore be different from the burden of disease associated to *Cryptosporidium spp* as a whole.

**Figure S2. Outcome tree for *Cryptosporidium spp b*ase case**

**
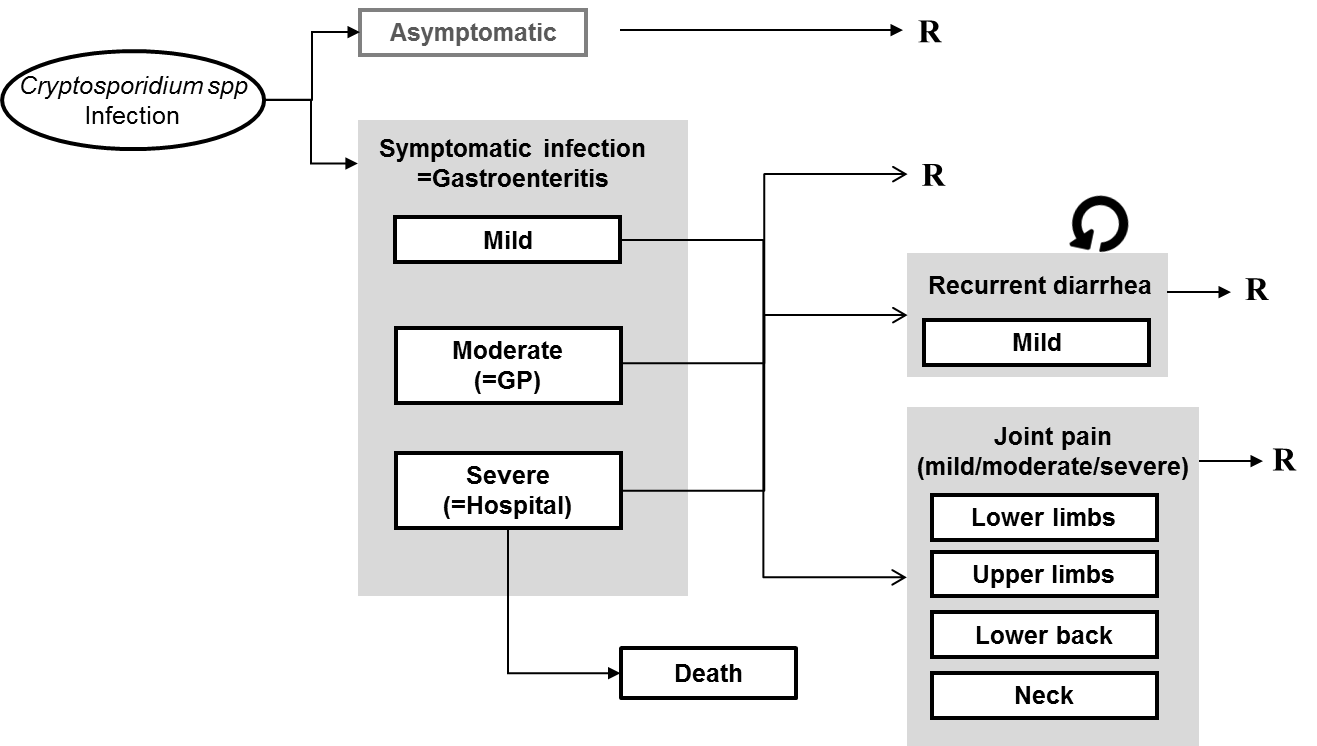
**

R: Recovery from health outcome; GP: General Practitioner

There is evidence that immunosuppressed patients experience higher frequency of cryptosporidiosis and a more severe disease course (15, 25, 28-30). However, for simplicity reasons we decided not to account for this in the models, assuming that this proportion of cases will be adequately represented by the population averages if the contexts are comparable. In the case of estimates from years before 1997, when the highly active antiretroviral therapy became widely available (31), we considered that the proportion of immunosuppressed individuals in the population would not be comparable to the current context, where people living with HIV are universally started on antiretroviral therapy (32).

As a final assumption, we did not consider that there was any disease burden derived from asymptomatic infections. Although some studies in low and middle-income countries have shown impaired growth in children with previous exposure to *Cryptosporidium* (27), this is not sufficiently established and likely not applicable to high-income countries with a low incidence such as the Netherlands.

***Input data for the model***

**Table S2** shows all the parameters used to build the model, data sources and the final input used in the model, along with the sensitivity and scenario analysis carried out.

*Incidence in the community*

We used **incidence data** provided by the Dutch *cryptosporidium* surveillance system. This is based on a sentinel network of laboratories voluntarily reporting weekly on *Cryptosporidium* cases, which is further corrected by coverage to provide national estimates. Annual number of cases is shown in **Table S3**. These cases were then assigned **by species** to either *C. parvum* or *C. hominis* following their proportional share based on data reported to the Dutch surveillance system. As this proportion varied greatly throughout the study period, as a base case we used for each year the share between species observed on that year (or the average for years with no surveillance data). As a sensitivity analysis we used a fix proportion using the average or the most extreme annual values.

Age distribution of cases in Dutch surveillance was significantly different between species, and also different from the one reported by Cassini et al.(5), based on European Surveillance data. We used the age distribution from Dutch surveillance. In the base-case we used the species-specific age-distribution, and in a sensitivity analysis we used the overall age distribution, including cases with unknown species. In the European data used by Cassini et al. (5) there were differences **by sex** in the age-group specific incidence of *Cryptosporidium*. However, since there is no information on differences by sex for most of the outcomes, and the life expectancy was taken from the Global Burden of Disease (GBD) 2010 study (33) that does not differentiate by sex, for simplicity reasons we did not account for sex differences, nor estimated the gender-specific burden of disease.

Finally, to estimate the **community incidence** from reported cases, we used as most likely multiplication factor (MF) 27.9. This was based on the second study of infectious intestinal disease (IID2) study from the UK (23), where they estimated a median of 8.2 (95% CI 2.1 to 31.7) community cases for each reported case. However, we considered that MFs in our context would be several times higher. In the UK *Cryptosporidium* is notifiable while in the Netherlands is not, gastroenteritis (GE) consultation and diagnosis rates are lower in the Netherlands than in the UK (26), and the multiplication rates in the IIED study excluded imported cases (which make up to 15% of all cases with available information in the Netherlands). A study found overall consultation rates in people with gastrointestinal symptoms in the Netherlands in 1996-1999 to be 5% after standardization by age and cohort, as compared to 17% in England (26). Therefore we considered our MFs would be 3.4 times higher than the ones reported by the IID2 study. We used a Pert-distribution to account for the uncertainty of the estimate, using as minimum and maximum 3.4 times the lower and upper bounds of the confidence interval from the IID2 study.

We further considered that this MF could vary by age. Normally GE in children can be more severe, with higher risk of dehydration and motivate more frequently seeking medical help. According to a Dutch study, consultation rates due to GE were around 2% for adults (18-64 years) (26). Comparted to adults, consultation rates were around 8 times higher for children 0 years, 4 times higher for 1-4 years, 3 times higher for 5-17 years and 4 times higher for those ≥65 years (26). Therefore, we assumed underascertainment was higher in adults (18-64 years). Compared to adults, we used a MF 87.5% lower in children 0 years, 75% lower for 1-4 years, 66.7% lower in children 5-14 years and 75% lower in ≥65 years.

However, according to expert opinion, consultation rates for GE cases may be more similar nowadays between the Netherlands and the UK, so as sensitivity analyses we assumed MFs would be just double the ones found in the IID2 study. We also considered in a sensitivity analysis that they did not vary by age.

*Number of cases by severity of the acute episode*

**Mild cases** of disease were considered not to request any medical help and were calculated as the number of cases in the community minus the number of cases that consulted a GP, assuming no deaths or hospitalizations occurring among mild cases.

Cases visiting a GP that were not hospitalized or deceased were considered **moderate cases**. To obtain the number of GP consultations, we applied the specific MFs from the IID2-study (23) that estimate a median of 2.3 GP consultations (95% CI 1.0 to 5.6) for each case captured by surveillance. As in the MFs for the community incidence, we believed these should be higher in the Netherlands as compared to the UK due to it not being a notifiable disease and the exclusion of imported cases. However, because consultation rates are lower in the Netherlands than in the UK (26), we only assumed MFs would be two times higher. As a sensitivity analysis we assumed they would be similar. We introduced these parameters in a Pert-distribution to account for the uncertainty of the estimate. As a base case, we assumed they would vary by age, using similar assumptions as the ones used for the MFs for community incidence. As sensitivity analysis we assumed constant MFs across age groups.

Hospitalized cases were considered **severe cases**. There were a number of studies reporting on hospitalization rates of *Cryptosporidium*, normally among laboratory confirmed cases or cases reported to surveillance. As the most likely value in our context, we used the hospitalization rate of 6.3% from a Dutch study (15). It was also the lowest estimate in the literature, so it was used as most likely value, and also as the minimum for a Pert distribution. Most of the other studies provided estimates around 14-15%, and up to 15.7%, which was used as the maximum. An estimate of 23% hospitalization rate was much higher than the rest of studies and was not used (1). Previous studies (18) have accounted for a 50% underreporting of hospitalization, therefore applying a MF of two. A recent report of a Swedish outbreak that reviewed hospital records for lab-confirmed cases found a hospitalization rate due to *Cryptosporidium* of 38.6% (25). Comparing this with the rate reported in Swedish surveillance of 15% (17), and assuming the differences were due to underreporting of hospitalization to the surveillance system, provided a MF of 2.6 for hospitalization rates. We applied a Pert distribution to account for uncertainty in the underreporting of hospitalization with 2.6 as most likely value, 2 as minimum and 3.2 as maximum, with no variation by age group. We assumed these MFs varied by age in the same way as described for MFs for community incidence and GP consultations, but in a scenario analysis we assumed they would be constant across age groups. Because there were no reports regarding ICU admissions due to cryptosporidiosis we assumed there was none.

Two valid estimates of the **mortality rate** (of 0% and 0.5%) for laboratory-confirmed cases were found in the literature. Other estimates had been calculated in contexts where a significant number of cryptosporidiosis was among HIV positive patients with high degree of immunosuppression (8, 11), and were considered not applicable to the current situation in the Dutch general population. The estimate of 0.5% by Mead et al. (18) is also old and probably reflects higher mortality than in the current scenario, while the study by Widerstrom et al.(25) that reported zero fatalities was too small to detect a single fatal case with mortality rates in the range reported by Mead et al. Because we believe in our current context mortality due to *Cryptosporidium* will be extremely low (if any), we used a Pert distribution with 0% as minimum and most likely value and 0.5% of laboratory-confirmed cases as a maximum. We then used the same MFs that we used to account for underreporting of hospitalization (i.e. Pert distribution with 2.6 as most likely and minimum of 2 and maximum of 3.2) and applied them for the underreporting of deaths, as was assumed in previous studies (18). As a scenario analyses we considered *Cryptosporidium* could not result in death.

To assign the fatal cases to the two species and the different age groups we first divided the estimated number of deaths proportionally between the two species (using the same parameters as described above). Then, we used the age distribution of fatal cases as the probability that a fatal case was in each age category and applied a discrete distribution to assign each fatal case to an age category. We performed this procedure separately for *C. hominis* and *C. parvum*, but assuming a similar age distribution of fatal cases between the two. As base case we assumed the age distribution of deaths with main cause of death registered as ICD-10 code A09 “Diarrhea and gastro-enteritis of presumed infectious origin”, extracted from CBS (Statistics Netherlands) for years 2014-2017 (34). As sensitivity analysis we used the age distribution reported by Cassini et al. (5) based on deaths reported in TESSy among *Cryptosporidium* and *Giardia* cases, in which deaths occurred at younger ages.

To determine life expectancy, we used the estimates from the GBD 2010 study for the base case (33), but as a sensitivity analysis we applied Dutch life expectancy estimates (35).

*Duration of the acute episode*

There was contradictory evidence regarding duration of disease **by species**, therefore we assumed similar duration between *C. hominis* and *C. parvum*. We used the **point estimates** of the studies and disregarded the measures of variability assuming that the variability would reflect the heterogeneity in the population and the duration would be adequately represented by the population average.

Mean days with diarrhea at the population level (including cases with complaints who were not laboratory confirmed) were found in different outbreak reports, ranging from 4.7 (6) to 9 days (9), with the most likely value set at 6.5 days (3). We used a Pert-distribution using those parameters to represent the duration in **mild cases**, although this could be an overestimation, since the study could include patients with moderate or severe disease. A study estimating 3 days (19) had very small sample size and was discarded.

Mean days with diarrhea in lab-confirmed cases (assumed to be moderate or severe) were reported by different sources. One study reported only duration up to the moment of requesting medical help (6), a follow-up study had low response rate and probable selection bias (22) and another study reported only categories(17), and were therefore disregarded. We used a uniform-distribution between the estimates of the remaining two studies, of 12.7 (13) and 16.7 days (15) (i.e. minimum and maximum) to represent the duration of **moderate cases**. This could also be an overestimation, since the studies population could include severe cases with longer duration.

The duration of the acute episode for **severe cases** was represented by hospitalized patients. A Swedish study (25), estimated mean durations of 24.5 days in immunosuppressed patients versus 11.2 in otherwise healthy people. A study in France found 30.6% of diagnosis in hospital laboratories were immunocompetent individuals (24). Applying this proportion to the above durations in hospitalized patients resulted in an average of 20.4 days of diarrhea that was used as a point estimate.

*Incidence, severity and duration of recurrent gastrointestinal symptoms*

We found evidence of recurrent gastrointestinal symptoms for both species (13, 14, 20, 22), and for all degrees of severity in the acute episode. To account for recurrent gastrointestinal symptoms, the frequency of recurrent **diarrhea** was generally considered. Other symptoms such as abdominal pain (13, 14, 20, 22), loss of appetite (13, 14), weight loss (13, 14, 22), nausea (20) or recurrent vomiting (13) were also demonstrated to occur after the acute episode, but the overlap between patients with multiple symptoms was generally not provided. Only Hunter et al. (13) reported on the proportion of patients with any gastrointestinal symptom. There was no evidence of differences **by species** in frequency of recurrent episodes

Studies in adults with laboratory-confirmed cryptosporidiosis (therefore **moderate and severe cases**) including a comparison group have found recurrence rates of between 9.1% (41.9% after illness vs. 32.8% before illness) (14) and 19.1% (24.3% in cases versus 5.2% in controls) (13), and a study based on Swedish surveillance estimated 15% (17). We used a Pert distribution with those parameters to account for the uncertainty (Pert-distribution (9.1%; 15%; 19.1%)). As a sensitivity analysis, we used the estimate of overall recurrent gastrointestinal symptoms, not only diarrhea, reported by Hunter et al. (13): 27.5% (40.9% vs. 13.4% in non-cases), as a point estimate.

To estimate the incidence of recurrent diarrhea in **mild cases**, we used data from an outbreak that reported different incidence rate in patients with clinical infection (21%) or laboratory-confirmed infection (39%) (7). Using the ratio between these two figures, we considered that incidence in mild cases would be 46% lower than the one in reported based on laboratory-confirmed cases. However, because the evidence was not strong, in a sensitivity analysis we considered that mild cases would experience the same incidence of recurrent diarrhea than moderate and severe cases.

There was no direct evidence of differences in incidence **by age**, but some studies suggested they could exist and were introduced as a scenario analysis. Two studies from the same outbreak using the same case definition for recurrent diarrhea found 52.4% in children <15 years of age (2) and 49.1% for adults (3). Based on this, we set that rates in children increased by 6.7% compared to the ones reported in adults, for all categories of severity.

There was little information on the **number of recurrent diarrhea episodes**. A study in adults showed that the number of patients developing one versus more than one recurrent episode of diarrhea was 1:1 among women and 2:1 among men (3). In a conservative approach, we assumed only 2 episodes for those reporting more than one episode and calculated that on average female patients experienced 1.5 episodes of recurrent diarrhea and male patients 1.3 episodes. As we do not distinguish between sex, did we use the average of 1.4. In children <15 years a rate of 3:1:0.7 had one, two or three or more relapses (2), therefore we assumed that children declaring 3 or more episode had exactly 3 and calculated 1.5 relapses per child with recurrent diarrhea on average. Scenario analyses were done considering double that number of episodes.

**Duration of recurrent episodes** was only reported by one study at a median of 2 days (range 1-15 days)(7). Because the mean in this study is not reported, we used Pert distribution with those values as most likely, minimum and maximum to obtain a better estimate of the duration. This was considered by the expert panel rather short, so in a sensitivity analysis two times this duration was considered. There was little information on **severity** of recurrent diarrhea episodes and, given the relatively short duration reported, in the base case we assumed they were all mild. In a scenario analysis we assumed a similar share of mild, moderate and severe episodes in recurrent episodes as in the acute episode.

*Incidence, severity and duration of joint pain*

**By species**, of the three studies reporting on joint pain after a follow-up period and with a control group (13, 14, 20), one reported only results for *C. hominis* (20), one found no difference between the two species (14), both having more joint pain after as compared to before the infection, and a third study found an association for *C. hominis* but not for *C. parvum* (13). A fourth study including only *C. parvum* (22) reported 33% patients with joint pain 6 or 12 months after the acute episode that was not present before; however, due to the longer follow-up, the low response rate and the absence of a control group, the quality of evidence was deemed insufficient. Because the evidence for *C. parvum* was lower, in the case base we considered it led to joint pain, but in a scenario analysis we considered it did not.

A Dutch study (14) reported **incidence of joint pain** of 6.5% (16.9% after illness vs. 10.4% before illness). Other studies had estimates of between 3% (11% in cases vs. 8% in controls)(20) and 10.7% (17.9% in cases vs. 7.2% in controls)(13). Therefore these values were taken as the most likely (6.5%), minimum (3%) and maximum (10.7%), respectively, using a Pert distribution. We run a scenario analysis in which children did not develop joint pain, since studies on reactive arthritis following *Campylobacter* infection did not find any case in children (36).

We did not find any analysis looking for the association between the **severity of the acute episode** and frequency of sequelae, therefore, incidence was assumed to be similar among mild, moderate and severe cases. However, as a scenario analysis we considered that mild cases would not develop joint pain as sequelae.

There was only one study (13) reporting on the **severity of the joint pain**, so the reported percentages of mild, moderate and severe pain were taken as point estimates, and assumed similar for any location of the pain. Regarding the **location** of the affected joint, we considered the simple average of the two available sources (13, 14) for the proportion of location in lower limbs, upper limbs, neck or back. Finally, also only one study looked at **duration** of the pain after 2 months of follow-up after the acute episode (13), and the estimate of 10.7 days was taken as a point estimate. A longer duration was assumed in a sensitivity analysis as studies reporting on duration of reactive arthritis after infection by other pathogens have found duration of symptoms of around 60 days (37) (36).

*Other long-term manifestations and sequelae*

Several studies have reported on dizziness (13, 14, 22) and fatigue (13, 14, 20, 22) as long-term manifestations after a *Cryptosporidium* infection. Since the nature and severity of these symptoms is not reported, there is no specific disability weight to account for these health states. They were included into the model only in a scenario analysis. Only estimates of incidence could be found, while there were no estimates of duration. A similar duration as for joint pain was assumed in absence of better data. Incidence of both dizziness and fatigue were taken as a uniform distribution between the estimates from the two studies with a control group (13, 14).

Other sequelae such as recurrent headaches (13), eye pain (13, 22), or irritable bowel syndrome (22) were not consistently reported and we considered they were not sufficiently proved.

*Model restrictions*

There were multiple parameter estimations via random distributions, especially for MFs to correct for underascertainment and underreporting for different outcomes, where Pert distributions were used to randomly select alternative values in Latin Hypercube iterations. In order to ensure that the simulations were internally consistent, we introduced correlations between these distributions. For the MFs for incidence in the community, visiting a GP, hospitalization and mortality, we introduced a correlation of 0.75 between the respective Pert distributions, meaning that the values from the respective distributions would be obtained from a similar range of the distribution (for example, all from the upper bound or all from some intermediate position in the distribution), while allowing for some variability.

*Discounts*

DALYs were discounted at 1.5%, according to the Dutch guidelines for health economic evaluation (38).

**Table S2. Input data for the *Cryptosporidium spp* burden of disease model**

| **Model parameter** | **Data source** | **Data extracted** | **Base case** | **Scenario and sensitivity analyses** |
| --- | --- | --- | --- | --- |
| Proportional share between species | Nic Lochlainn et al. 2018 (15): both species | Data reanalyzed for the full database (not restricting to responders of the case-control study questionnaire).  - Overall: *C. parvum* (31.52%), *C. hominis* (14.66%) and unknown/other species (53.82%). Thus, of those with known species: *C. parvum* (68.25%), *C. hominis* (31.75%)  - Most extreme annual proportions:  *C. parvum* (87.32%), *C. hominis* (12.68%)  *C. parvum* (51.75%), *C. hominis* (48.25%) | 2013: *C. hominis* = 19.43%  2014: *C. hominis* = 12.68%  2015: *C. hominis* = 48.25%  2016: *C. hominis* = 73.68 %  2017: *C. hominis* = 31.75% (the average) | *- C. hominis* 31.75% for all years  - *C. hominis* 12.68% for all years  - *C. hominis* 48.25% for all years |
| Age distribution (among lab-confirmed cases) | Nic Lochlainn et al. 2018 (15): both species, separately  Data reanalyzed for the full database (not restricting to responders of the case-control study questionnaire). Overall includes also patients with unknown species. | \| Age \| Hominis (%) \| Parvum (%) \| Overall (%)(15)(15)(15)(15)(15)(15)(15)(15) \| \| --- \| --- \| --- \| --- \| \| 0 \| 14.93 \| 8.24 \| 10.74 \| \| 1-4 \| 20.49 \| 15.99 \| 17.92 \| \| 5-9 \| 21.18 \| 12.44 \| 17.36 \| \| 10-14 \| 6.94 \| 4.20 \| 4.99 \| \| 15-19 \| 2.78 \| 5.33 \| 4.12 \| \| 20-24 \| 1.74 \| 3.88 \| 4.07 \| \| 25-29 \| 3.82 \| 6.79 \| 5.24 \| \| 30-34 \| 6.60 \| 7.11 \| 6.52 \| \| 35-39 \| 7.29 \| 5.82 \| 6.77 \| \| 40-44 \| 2.43 \| 6.95 \| 5.04 \| \| 45-49 \| 1.39 \| 5.82 \| 3.72 \| \| 50-54 \| 2.43 \| 4.04 \| 3.21 \| \| 55-59 \| 3.47 \| 3.23 \| 2.60 \| \| 60-64 \| 1.74 \| 3.39 \| 2.90 \| \| 65-69 \| 2.08 \| 1.94 \| 2.14 \| \| 70-74 \| 0.69 \| 2.10 \| 1.07 \| \| 75-79 \| 0.00 \| 1.29 \| 0.87 \| \| 80+ \| 0.00 \| 1.45 \| 0.71 \| | Age distribution different by species | Overall age distribution (including cases with unknown species) assumed for both species |
| Incidence in the population | Tam et al. 2012 (23): not specified, probably only *C. parvum* | For each case reported to national surveillance there are a median of 8.2 (2.5th and 97.5th centiles: 2.1-31.7) cases in the community | Pert distribution:  Most likely = 27.9  Minimum = 7.1  Maximum = 107.8  Varying MFs by age with the following modifiers:  0 years=0.125 times the MF  1-4 years=0.25 times the MF  5-14 years=0.333 times the MF  15-64= reference  >=65 years=0.25 times the MF  Number of cases was calculated to respect the relative difference between age groups while keeping the overall MF constant.  Assumed similar between species | - MFs only double the ones in the UK, Pert distribution:  Most likely = 16.4  Minimum = 4.2  Maximum = 63.4  -MFs are constant across age-groups |
|  | De Wit et al. 2001 (26) | Overall GP consultation rate in patients with gastrointestinal symptoms = 5% after standardization by age and cohort (compared to 17% in England)  Consultation rates for the different age groups:  0 years=15.9%  1-4 years=8.3%  5-11 years = 5.9%  12-17 years=6.7%  18-64= 2.0%  >=65 years=8.3% |  |  |
| Cases presenting to the GP | Tam et al. 2012 (23): not specified, probably only *C. parvum* | For each case reported to national surveillance there are 2.3 (1-5.6) cases presenting to the GP | Pert distribution:  Most likely = 4.6  Minimum = 2.0  Maximum = 11.2  Varying MFs by age with modifiers similar to the ones above for incidence in the population  Assumed similar between species | - MFs are the same as in the UK, Pert distribution:  Most likely = 2.3  Minimum = 1.0  Maximum = 5.6  -MFs are constant across age-groups |
|  | De Wit et al. 2001 (26) | Overall consultation rate in patients with GE = 5% after standardization by age and cohort (compared to 17% in England)  GP consultation rates in GE patients by age:  0 years=15.9%  1-4 years=8.3%  5-11 years = 5.9%  12-17 years=6.7%  18-64= 2.0%  >=65 years=8.3% |  |  |
| Hospitalization rate (among lab-confirmed cases) and MFs | Nic Lochlainn et al. 2018 (15):  overall | 6.3%, no difference by species | Hospitalization rate (applied to cases in surveillance), Pert distribution:  Most likely = 6.3%  Minimum = 6.3%  Maximum = 15.7%  MFs for underreporting,  Pert distribution:  Most likely = 2.6  Minimum = 2  Maximum = 3.2  Varying MFs by age with modifiers similar to the ones above for incidence in the population | - MFs are constant across age-groups |
|  | Stiff at al. 2017 (22): *C. parvum* | 11.1% |  |  |
|  | Abal-Fabeiro et al 2015 (1): overall | 23.0%, higher for *C. parvum* (but differences disregarded) |  |  |
|  | Dietz et al. 2000 (8): *C. parvum* | 15.7% |  |  |
|  | Widerstrom et al. 2011 (25): *C. hominis* | 63 hospitalizations (7 for a different reason) in an outbreak with 145 lab-confirmed cases (unclear if it’s the same patients) |  |  |
|  | Hunter at al 2004 (12): both species | 14% |  |  |
|  | Insulander et al. 2013 (17): overall | 15% |  |  |
|  | Mead et al. 1999 (18): all species | 0.150 (15.0%). MF of 2 for underreporting |  |  |
|  | De Wit et al. 2001 (26) | GP consultation rates in GE patients by age:  0 years=15.9%  1-4 years=8.3%  5-11 years = 5.9%  12-17 years=6.7%  18-64= 2.0%  >=65 years=8.3% |  |  |
| Duration of acute episode of diarrhea (overall population) | Widerstrom 2014 (3): *C. hominis* | Overall: median 4 days (range 1–51 days)  Calculated mean (based on data reported in a Figure): 6.5 days  Significant differences by age group | Duration of acute episode in mild cases =  Pert distribution:  Most likely: 6.5 days  Minimum: 4.7 days  Maximum: 9 days  Variation by age assumed to be accounted for when using the population average |  |
|  | Adler et al. 2017 (2): *C. hominis* | Children <15 years: mean 7.5 days (median 6, range 1–80 days). In 13.2% lasting ≥14 days, and in 2% lasting ≥30 days |  |  |
|  | Corso et al. 2003 (6) : C. parvum | Mild illness: mean of 4.7 days before seeking medical help (in the case of mild cases represents total duration) |  |  |
|  | Ethelberg et al. 2009 (9): *C. hominis* | 9 days (range 2–31 days) |  |  |
|  | Insulander et al. 2008 (16): *C. parvum* | ≥ 7 days for 86%  (Small sample size) |  |  |
|  | Mc Cann et al. 2014 (19): *C. hominis* | median 3 days (range 1–9 days, mean 3.75 days)  (Small sample size) |  |  |
| Duration of acute episode of diarrhea (among lab-confirmed cases) | Nic Lochlainn et al. 2018 (15): overall | Median 13 days (range 1-64) , mean (self-calculated): 16.7 longer for *C. hominis*, but difference not accounted for due to contradictory evidence | Duration of acute episode in moderate cases =  Uniform distribution:  Minimum: 12.7 days  Maximum: 16.7 days |  |
|  | Stiff at al. 2017 (22): *C. parvum* | Median 23 days (range 7 – 84 days) (low response rate, small sample) |  |  |
|  | Hunter at al 2004 (12): both species | Mean of 12.7 days and median of 11 days |  |  |
|  | Corso et al. 2003 (6) : C. parvum | Moderate illness: mean of 5.8 days before seeking medical help (underestimation of total duration) |  |  |
|  | Insulander et al. 2013 (17): both species | 1-3 days (4%), 4-10 days (27%) and >10 days (69%). Longer duration for *C. parvum* but not accounted for due to contradictory evidence |  |  |
| Duration of acute episode of diarrhea (hospitalized patients) | Widerstrom et al. 2011 (25): *C. hominis* | Mean in immunosuppressed longer compared with non-immunosuppressed (24.5 versus 11.2 days) | Point estimate= 20.4 |  |
|  | The ANOFEL *Cryptosporidium* National Network 2010 (24) | 114/372 (30.6%) cases diagnosed of *Cryptosporidium* with known immune status were immunocompetent |  |  |
|  | Corso et al. 2003 (6) : C. parvum | Moderate illness: mean 18.4 days before seeking medical help |  |  |
| Age distribution of deceased cases | Cassini et al. 2018 (5): both species | \| Age \| Cassini et al (5)(%) \| CBS (34)(%) \| \| --- \| --- \| --- \| \| 0 \| 12.5 \| 0.12 \| \| 1-4 \| 6.25 \| 0.06 \| \| 5-9 \| 6.25 \| 0.19 \| \| 10-14 \| 0 \| 0.06 \| \| 15-19 \| 0 \| 0 \| \| 20-24 \| 0 \| 0.12 \| \| 25-29 \| 0 \| 0.06 \| \| 30-34 \| 6.25 \| 0.19 \| \| 35-39 \| 0 \| 0.06 \| \| 40-44 \| 0 \| 0.12 \| \| 45-49 \| 6.25 \| 0.74 \| \| 50-54 \| 12.5 \| 0.74 \| \| 55-59 \| 6.25 \| 1.67 \| \| 60-64 \| 6.25 \| 2.17 \| \| 65-69 \| 6.25 \| 4.15 \| \| 70-74 \| 6.25 \| 5.88 \| \| 75-79 \| 18.75 \| 9.60 \| \| 80-84 \| 6.25 \| 17.34 \| \| 85-89 \| 24.58 \| \| 90-94 \| 22.35 \| \| 95+ \| 9.78 \| | Age distribution of deaths assumed as in CBS data (34) | Age distribution of deaths assumed as in Cassini et al. (5) |
|  | CBS (Statistics Netherlands)(34) years 2014-2017. |  |  |  |
|  |  |  |  |  |
| Mortality rate (in lab-confirmed cases) | Widerstrom et al. 2017 (25): *C. hominis* | 0% (small sample size) | Pert distribution (applied to cases in surveillance):  Most likely=0%  Minimum=0%  Maximum=0.5%  Pert distribution for MFs underreporting similar to the ones for hospitalization:  Most likely = 2.6  Minimum = 2  Maximum = 3.2 | Mortality rate = 0% |
|  | Mead et al. 1999 (18): all species | 0.005 (0.5%)  MF of 2 for underreporting. |  |  |
| Incidence of recurrent diarrhea (among lab-confirmed cases) | Igloi et al. 2018 (14): both species | 41.9% after illness vs.32.8% before illness. | Pert distribution for incidence of recurrent diarrhea when the acute episode was moderate or severe:  Most likely = 15%  Minimum = 9.1%  Maximum = 19.1% | -Rates 6.7% higher in children (result of the Pert distribution increased by 6.7% for children <15 years).  -MFs in adults down-weighted proportionally  - Extreme scenario using the rates of overall gastrointestinal recurrent symptoms reported by (13): 27.5% |
|  | MacKenzie et al 1995 (7): *C. parvum* | Overall 32%, in lab confirmed-cases 39%. |  |  |
|  | Hunter at al 2004 (13): both species | 24.3% diarrhea vs. 5.2% of controls.  40.9% one of vomiting, diarrhea, loss of appetite or abdominal pain vs. 13.4% of controls. |  |  |
|  | Insulander et al. 2013 (17): both species | 15% intermittent diarrhea at 25-36 months of follow-up, with no difference between species. At 6-12 months was 10%. |  |  |
| Number of recurrent episodes in those with recurrent diarrhea | Widerstrom 2014 (3): *C. hominis* | Overall 49.1%.  Number of episodes: among women, 1 relapse 25.4%, >1 relapse 24.1%; among men, 1 relapse 33.5%, > 1 relapse 15.0%. | Number of episodes:  1.4 in adults  1.5 in children |  |
|  | Adler et al. 2017 (2): *C. hominis* | Children <15 years: Overall 52.4%  Number of episodes: 33.5% one relapse, 11.2% two relapses, and 7.8% ≥3 relapses |  |  |
| Incidence of recurrent diarrhea (overall population) | MacKenzie et al. 1995 (7): *C. parvum* | Incidence of 21% in people with clinical infection (vs. 39% in lab confirmed-cases) | Incidence of recurrent diarrhea when the acute episode was mild: 46% lower than the one for moderate and severe cases | -Similar incidence by severity  - Double number of episodes |
|  | Rehn et al. 2015 (20): *C. hominis* | Diarrhea 18% vs. 6% of controls in location 1 and 17% vs. 5% of controls in location 2. |  |  |
| Duration of a recurrent episode | MacKenzie et al. 1995 (7): *C. parvum* | Median 2 days (range 1-15 days), mean is not reported. | Pert distribution:  Most likely = 2  Minimum = 1  Maximum = 15 | Double that duration |
| Incidence of joint pain | Igloi et al. 2018 (14):both species | 16.9% after illness vs.10.4% before illness | Pert distribution:  Most likely =6.5%  Minimum = 3%  Maximum = 10.7% | *-C.parvum* does not cause joint pain  - Mild cases do not result in joint pain  - Children do not develop joint pain. |
|  | Hunter et al. 2004 (13): both species | 18.0% of *C. hominis,* 12.0% of *C.parvum*, 17.9% overall vs. 7.2% of controls (not statistically significant) |  |  |
|  | Rehn et al. 2015 (20): *C. hominis* | 11% in cases vs. 8% in non-cases in location 1  14% in cases vs. 8% in non-cases in location 2 |  |  |
|  | Hannu et al. 2002 (36) | Campylobacter-related reactive arthritis (of 609 Campylobacter-positive patients): incidence of 9% in adults and 0% in children. |  |  |
| Severity of the joint pain | Hunter at al. 2004 (13): both species | Mild= 39%  Moderate = 53%  Severe = 8% | Proportions taken from (13), assumed similar for all locations |  |
| Localization of the joint pain | Igloi et al. 2018 (14):both species | Lower limbs 46%  Upper limbs 38%  Back 4%  Neck 12% | Location as mean of the two studies:  Lower limbs 43%  Upper limbs 36%  Back 14%  Neck 9% |  |
|  | Hunter at al. 2004 (13): both species | Lower limbs 39%  Upper limbs 33%  Back 23%  Neck 5% |  |  |
| Duration of the joint pain | Hunter et al. 2004 (13): both species | Mean 10.7, standard deviation 12.7 days, median 5.0 days | Point estimate = 10.7 days | - Point estimate = 60 days |
|  | Hannu et al. 2002 (36) | Duration assessed in 20 patients with *Campylobacter*-related reactive arthritis: 10 patients ≤1 month, 3 patients 1-2 months, 1 patient 2-3 months, 5 patients 3-4 months, 1 patient 4-6 months (average duration taking mid-points 52days) |  |  |
|  | Locht et al. (37) | Duration of joint symptoms of reactive arthritis following Campylobacter infection, median 60 days |  |  |
| Incidence of dizziness | Stiff at al. 2017 (22): *C. parvum* | Dizzy spells (10 %) at 6 or 12 months, low response rate | No dizziness as long-term manifestation | Uniform distribution:  Minimum: 2.5 %  Maximum: 7.1 % |
|  | Igloi et al. 2018 (14):both species | 14.6% after illness vs. 7.5% before illness |  |  |
|  | Hunter at al. 2004 (13): both species | Overall, 15/235 (6.4%) in cases vs. 9/232 in controls (3.9%)  8/61 (13.1 %) for *C. hominis* and 2/50 (4 %) for *C. parvum* |  |  |
| Incidence of fatigue | Stiff at al. 2017 (22): *C. parvum* | Fatigue (22 %) at 6 or 12 months, low response rate | No fatigue as long-term manifestation | Uniform distribution:  Minimum: 11.8 %  Maximum: 16.6 % |
|  | Igloi et al. 2018 (14):both species | 50.7% after illness vs.34.1% before illness |  |  |
|  | Hunter at al. 2004 (13): both species | Overall, 45/235 (19.1%) in cases vs. 17/232 (7.3%) in controls  16/61 (26.2 %) for *C. hominis* and 8/50 (16 %) for *C. parvum* |  |  |
|  | Rehn et al. 2015 (20): *C. hominis* | 26% in cases vs. 16% in controls in location 1  27% in cases vs. 14% in controls in location 2 |  |  |

MF: Multiplication Factors, GE: Gastroenteritis, GP: General Practitioner

**Table S3. Number of cases in the Dutch C*ryptosporidium spp* surveillance system**

| **Year** | **2013** | **2014** | **2015** | **2016** | **2017** |
| --- | --- | --- | --- | --- | --- |
| Number of cases | 973 | 988 | 1769 | 2108 | 1332 |

# Disability Weights

The disability weights (DW) were taken from Haagsma et al.(39). The concept and the DW applied to each of the health outcomes in our study are shown in **Table S4**. For some of the outcomes for severe joint pain, no DW was available and, in those circumstances, it was assumed to be similar to the DW of the moderate pain. For neck pain, a DW for moderate, acute pain was not available and was assumed similar to the mild pain in a conservative approach. For dizziness and fatigue as sequelae of *Cryptosporidium*, since these did not fulfill the definition for chronic fatigue syndrome, we used the DW for Infectious disease, acute episode, mild (0.007).

**Table S4. Disability weights from Haagsma et al.(39) for each of the health outcomes of the model.**

| **Outcome in the model** | **Health state *** | **Description*** | **Disability weight*** |
| --- | --- | --- | --- |
| Symptomatic infection  (mild) | Diarrhea, mild | has diarrhea three or more times a day with occasional discomfort in the belly | 0.073 (0.061 - 0.092) |
| Symptomatic infection  (moderate) | Diarrhea, moderate | has diarrhea three or more times a day, with painful cramps in the belly and feeling thirsty | 0.149 (0.12 - 0.182) |
| Symptomatic infection  (severe) | Diarrhea, severe | has diarrhea three or more times a day with severe belly cramps. The person is very thirsty and feels nauseous and tired | 0.239 (0.202 - 0.285) |
| Recurrent / persistent diarrhea  (mild) | Diarrhea, mild | has diarrhea three or more times a day with occasional discomfort in the belly | 0.073 (0.061 - 0.092) |
| Recurrent diarrhea  (moderate) | Diarrhea, moderate | has diarrhea three or more times a day, with painful cramps in the belly and feeling thirsty | 0.149 (0.12 - 0.182) |
| Recurrent diarrhea  (severe) | Diarrhea, severe | has diarrhea three or more times a day with severe belly cramps. The person is very thirsty and feels nauseous and tired | 0.239 (0.202 - 0.285) |
| Joint pain in lower limbs (mild) | Musculoskeletal problems, lower limbs, mild | has pain in the leg, which causes some difficulty running, walking long distances, and getting up and down | 0.027 (0.021 - 0.032) |
| Joint pain in lower limbs (moderate) | Musculoskeletal problems, lower limbs, moderate | has moderate pain in the leg, which makes the person limp, and causes some difficulty walking, standing, lifting and carrying heavy things, getting up and down and sleeping. | 0.094 (0.08 - 0.12) |
| Joint pain in lower limbs (severe) | Musculoskeletal problems, lower limbs, severe | has severe pain in the leg, which makes the person limp and causes a lot of difficulty walking, standing, lifting and carrying heavy things, getting up and down, and sleeping. | 0.134 (0.11 - 0.165) |
| Joint pain in upper limbs (mild) | Musculoskeletal problems, upper limbs, mild | has mild pain and stiffness in the arms and hands. The person has some difficulty lifting, carrying and holding things | 0.041 (0.032 - 0.05) |
| Joint pain in upper limbs (moderate) | Musculoskeletal problems, upper limbs, moderate | has moderate pain and stiffness in the arms and hands, which causes difficulty lifting, carrying, and holding things, and trouble sleeping because of the pain. | 0.138 (0.114 - 0.167) |
| Joint pain in upper limbs (severe) | *Assumed similar to joint pain in upper limbs (moderate)* | - | - |
| Joint pain back (mild) | Low back pain, mild | has mild back pain, which causes some difficulty dressing, standing, and lifting things | 0.024 (0.018 - 0.03) |
| Joint pain back (moderate) | Low back pain, moderate | has moderate back pain, which causes difficulty dressing, sitting, standing, walking, and lifting things. | 0.060 (0.05 - 0.074) |
| Joint pain back (severe) | *Assumed similar to* *joint pain back (moderate)* | - | - |
| Joint pain neck (mild) | Neck pain, acute, mild | has neck pain, and has difficulty turning the head and lifting things | 0.062 (0.05 - 0.075) |
| Joint pain neck (moderate) | *Assumed similar to* *joint pain back (mild)* | - | - |
| Joint pain neck (severe) | Neck pain, acute, severe | has severe neck pain, and difficulty turning the head and lifting things. The person gets headaches and arm pain, sleeps poorly, and feels tired and worried. | 0.224 (0.19 - 0.268) |
| Dizziness as a long term manifestation | Infectious disease, acute episode, mild | has a low fever and mild discomfort , but no difficulty with daily activities | 0.007 (0.005 - 0.01) |
| Fatigue as a long term manifestation (not fulfilling definition for chronic fatigue syndrome) | Infectious disease, acute episode, mild | has a low fever and mild discomfort , but no difficulty with daily activities | 0.007 (0.005 - 0.01) |

# Costs

For the costs of an acute episode we made similar assumptions as those in Mangen et al. (40), with small modifications. Table S5 summarizes the sources and the assumptions used when they were extracted from particular studies not considered by Mangen et al. (40).

***Direct healthcare costs (DHC)***

**Mild cases** were assumed not to incur in any DHC.

There was no specific information regarding medical visits, so we assumed the same inputs as used in Mangen et al. (40) for **moderate GE cases**: 90% visited a GP, 10% had a house call from the GP, and there were 0.97 telephone consultations per GP visit (26). For moderate cases we assumed no specialized care outpatient visits. We assumed that about 27% would get a prescription for antibiotics, same as for overall GE (41), with azithromycin (since there paramomycin and nitazoxanide are not available in the Netherlands). A fecal sample was assumed to be taken in 18% of the cases, which is the proportion reported for overall GE in the Netherlands (21), with no differences by age (42). This may result in an underestimation, as *Cryptosporidium* can result in watery diarrhea, which is more frequently tested.

We assumed that **severe GE** patients younger than 18 years had 1.3 GP consultations during regular hours, 0.7 GP consultations in weekends/evening hours, 0.4 outpatient clinic visits and 0.1 emergency department visits. For persons ≥ 18 years it was assumed to be 1.7, 0.3, 1.4 and 0.3, respectively (43). About 27% get a prescription for antibiotics (41), and a fecal sample was assumed be taken in 100% of severe cases. About 1%, 19% and 71% of patients younger than 18 years, patients between 18-64 years and elderly patients (≥ 65 years), respectively would be transported to the hospital by ambulance (43).

For the mean duration of hospital stay we re-analyzed the data from a Dutch case-control study (15) by age group: 5.3 days for <5 years of age; 3.6 days for 5-14 years of age; 5.7 days for 15-64 years of age; and 7.0 days for ≥65 years of age. We assumed, according to the GEops study, that approximately 5.9% of the elderly non-fatal severe GE patients (≥ 65 years) would be transferred from hospital into a nursing home for some 60 days before returning home (43).

Where available, we used Dutch reference prices from the Dutch guidelines for health economic evaluation (38), and otherwise unitary costs were taken from Mangen et al (40, 44) and updated to 2017 Euros. This resulted in: antibiotics (including pharmacy costs, 4.09 €), GP consultation (including regular hours or evening/weekends, 33.78 €), GP house consultation (51.18 €), GP telephone consultation (17.4 €), outpatient clinic visit (93.15 €), emergency department visit (265.11 €), hospitalization per day (641.79 € in children and 487.23 € in adults), urgent transport by ambulance (627.46 €) and nursing home, per day (171.96 €). The unitary cost of a fecal sample laboratory test was updated to 62.73 € according to the Dutch health system public prices for 2017.

We assumed that **fatal cases** would incur the same DHC costs as non-fatal severe GE cases except for nursing home costs, as they would have died before being admitted to a nursing home.

There was no available data regarding GP consultation due to **recurrent diarrhea following acute cryptosporidiosis**. In the base case we assumed that the severity of episodes is mild and for estimation of costs we consider they did not motivate any medical consultations. However, it is plausible that repeated episodes of mild symptoms could trigger medical consultation and laboratory investigation, so as a scenario analysis we considered recurrent episodes of mild diarrhea would be similar to moderate acute episodes with regards to medical consultations, prescribed medication and laboratory tests. For moderate and severe episodes of recurrent diarrhea we assumed similar direct health care costs and patient costs as for the acute episodes of comparable severity, while other sector’s costs were corrected for the shorter duration of the recurrent episode.

For **joint pain**, we considered that 100% of severe cases, 41.5% of moderate cases and 0% of mild cases will visit a GP (13), assuming all were GP visits (no house calls or telephone calls). We assumed no hospital admissions or emergency department consultations due to joint pain. We assumed no diagnostic tests were performed for this condition.

**Dizziness** and **fatigue** were assumed to be of mild severity and not to incur in any DHC.

***Direct non-healthcare costs (DNHC)***

Assumptions for the **acute** GE epi**sodes** were similar to those published by Mangen et al.(40). Travel costs were considered for GP visits and in- and outpatient visits. When visiting a GP we assumed that 77% and 97% of the persons younger than 18 years and older than 18 years, respectively, would use a car/public transport (half/half), whereas in the case of hospitalization 97% of persons younger than 18 years would be transported to the hospital using a car/public transport (half/half), and 100% of the persons older than 18 years for simplicity (very small proportion will use an ambulance) (43). Regarding **over the counter medications (OCM)**, like Mangen et al.(40) we assumed that mild acute cases would use anti-diarrheal drugs (31%), ORS (5%) and painkillers (5%) as OCM. For moderate and severe acute cases we assumed that the corresponding values would be 59%, 33% and 5%, respectively (45, 46). We assumed that fatal GE cases would incur the same DNHC costs as non-fatal severe GE cases until their death, except transport to nursing homes.

**Recurrent episodes** of diarrhea were assumed to use similar OCM as acute GE episodes. For cases of **joint pain** we used the GP consultation rates calculated based on Hunter et al. (13) to make our assumption about use of OCM medication (anti-inflammatory drugs and pain-killers), and considered that the proportion of mild cases using these drugs would be 0%, 41.5% and 100%, respectively for mild, moderate and severe cases. We considered they used these medications during half of the total duration of a joint pain episode (i.e. 5,35 days).For **dizziness** and **fatigue** we did not consider patient costs, since there were no transports to consultations and no evidence on OCM to account for.

We used the unitary costs for OCM as used by Mangen et al(40) and updated to 2017 Euros, at: €1.36 and €2.54 for children and adults, respectively, with mild infection; and €4.41 and €8.19, for children and adults, respectively, with moderate to severe infection. The costs for additional diapers for children younger than 5 years would be €3.57, €7.13 and €7.61 for mild, moderate and severe acute cases, respectively (40), and half of that for a recurrent diarrhea episode. The cost for anti-inflammatory drugs and pain-killers for joint pain was estimated at €1.69 per case in 0-14 years of age and €1.89 for persons >14 years.

***Indirect non-healthcare costs (INHC)***

For the duration of hours of work (i.e. productivity) lost due to the **acute diarrhea**, there were some specific reports for *Cryptosporidium*. But because the average working hours are lower in the Netherlands as compared to other countries, we decided as a more conservative estimate, to use specific Dutch estimates for GE in general as used in Mangen et al (40), based in the GEops and Pienter 2 studies. We used similar number of hours of both paid and unpaid work lost due to mild, moderate and severe acute episodes for the patients and/or their caregivers. The price in euros for each hour lost in productivity by age group was also used as in Mangen et al. (40) and updated to 2017 Euros.

For **deceased persons** we used the friction costs approach, were loss of productivity is only accounted for during the estimated time until a replacement is found for that work position. The price for hour of productivity lost due to absence from work was the same as for sick leaves and the average number of working hours needed to find a replacement from the working position were also used as in Mangen et al.(40) and varied by age between 110.6 hours for a person 15-19 years to 333.7 hours for a person 45-49 years.

For **recurrent episodes of diarrhea**, we adjusted all loss of productivity proportionally to the duration of the recurrent episode relative to an acute episode. For loss of productivity due to **joint pain**, we used the assumptions used in Mangen et al (40). regarding post-infectious reactive arthritis, assuming as well that mild cases would not generate any loss of productivity and assuming joint pain following cryptosporidiosis, either moderate or severe, would be equivalent to moderate reactive arthritis (as we assumed no emergency department visits or hospitalization for severe cases of joint pain). Only loss of paid work was accounted, since there was no evidence of loss of unpaid work. For **dizziness** and **fatigue**, in absence of better data and since we had considered similar duration as a joint pain episode, we also considered similar productivity loss as a joint pain episode.

**Table S5. Input data for the *Cryptosporidium* costs model**

| **Model parameter** | **Data source** | **Data extracted** | **Base case** |
| --- | --- | --- | --- |
| Duration of hospitalization *Cryptosporidium* | Nic Lochlainn et al. 2018 (15): overall | Median 4 days (range 0-19), with no difference by species  Mean (self-calculated) = 5.3  By age group (self-calculated) :5.3 days for <5 years of age; 3.6 days for 5-14 years of age; 5.7 days for 15-64 years of age; and 7.0 days for ≥65 years | 5.3 days for <5 years of age  3.6 days for 5-14 years of age  5.7 days for 15-64 years of age  7.0 days for ≥65 years of age |
|  | Stiff at al. 2017 (22): *C.Parvum* | Mean 15.8 days SD±13.3; range 2–34 days (low response rate, small sample) |  |
|  | Corso et al. 2003 (6) : *C.Parvum* | If no underlying conditions: 5 days, 16 days if aids, 7 if other conditions, all conditions 8 days. Results in overall of 5.9 days if applying proportion of immunocompetents from Widerstrom et al(25) |  |
|  | Hunter at al 2004 (12): both species | Median 3 days (range 1–9) |  |
|  | Widerstrom et al. 2011 (25): *C.hominis* | Median 4 days (range 1-125) |  |
| Consultation rates for joint pain | Hunter at al. 2004 (13): both species | Mild= 39%  Moderate = 53%  Severe = 8%  7 cases (19%) and 2 controls (15%) had already consulted a doctor and 4 cases (11%) and 1 control (8%) were intending to do so. | 0% of mild cases  41.5% of moderate cases ((0.30-0.08)*0.53))  100% of severe cases |

# Detailed results of burden of disease and costs of illness

***Results in the base case***

Table S6 shows detailed results for years between 2013 and 2017.

***Results of scenario and sensitivity analyses***

Table S7 shows the overall estimates of burden of disease and cost of illness under 28 different scenario and sensitivity analyses. They have only been applied to year 2017, since the impact of all scenarios will be similar throughout the years.

**Table S6. Point estimation (and 95% Uncertainty Interval) for Disability Adjusted Life Years (DALYs) and costs of illness (COI) of *Cryptosporidium* infection in the Netherlands: base-case model for years 2013 - 2017.**

**Year: 2013**

|  | | | DALYs* | | | | Costs^#^ | | | | |
| --- | --- | --- | --- | --- | --- | --- | --- | --- | --- | --- | --- |
|  |  |  | YLD | YLL | Total DALYs | DALYs per 1,000 cases | DHC  (million €) | Patient costs (million €) | Productivity losses (million €) | Total costs (million €) | Costs per 1,000 cases  (*1,000 €) |
| **Overall** | |  | 83  (32-158) | 17  (0-64) | 100  (39-188) | 3  (2-5) | 1  (0.7-1.4) | 0.2  (0.1-0.3) | 12.5  (4.2-24.3) | 13.6  (5-25.9) | 384  (335-479) |
| **Per million inhabitants** | |  | 5  (2-9) | 1  (0-4) | 6  (2-11) | - | 0.06  (0.04-0.08) | 0.01  (0.01-0.02) | 0.7  (0.2-1.4) | 0.8  (0.3-1.5) | - |
| **Acute episode** | |  | 74  (29-140) | 17  (0-64) | 91  (35-171) | 2.7  (1.7-5.1) | 1  (0.7-1.4) | 0.1  (0.1-0.3) | 11.4  (3.9-22) | 12.5  (4.7-23.6) | 352  (311-442) |
| **Long-term manifestations** | **Diarrhea** | | 4  (1-11) | 0  (0-0) | 4  (1-11) | 0.1  (0.03-0.24) | 0  (0-0) | 0.01  (0-0.02) | 0.74  (0.13-2.28) | 0.8  (0.1-2.3) | 21  (6-50) |
|  | **Joint pain** | | 6  (1-12) | 0  (0-0) | 6  (1-12) | 0.15  (0.09-0.22) | 0.02  (0.01-0.06) | 0  (0-0.01) | 0.36  (0.1-0.82) | 0.4  (0.1-0.9) | 11  (6-15) |
|  | **Total** | | 9  (2-21) | 0  (0-0) | 9  (2-21) | 0.3  (0.1-0.4) | 0.02  (0.01-0.06) | 0.02  (0-0.03) | 1.11  (0.26-2.89) | 1.1  (0.3-3) | 31.6  (15.3-61.2) |
| **By species** | ***C. hominis*** | | 16  (6-31) | 3  (0-18) | 19  (6-39) | 2.8  (1.9-6.3) | 0.2  (0.1-0.3) | 0.03  (0.01-0.06) | 2.4  (0.8-4.7) | 2.6  (0.9-5) | 375  (331-461) |
|  | ***C. parvum*** | | 67  (26-127) | 14  (0-53) | 81  (32-152) | 3  (2-5.4) | 0.8  (0.5-1.2) | 0.13  (0.05-0.24) | 10.1  (3.4-19.7) | 11  (4.1-21) | 386  (336-484) |

*The DALYs are discounted at 1.5% per year according to the Dutch guidelines for health economics(38); ^#^costs are not discounted since all costs happened within the year of infection.

**Year: 2014**

|  | | | DALYs* | | | | Costs^#^ | | | | |
| --- | --- | --- | --- | --- | --- | --- | --- | --- | --- | --- | --- |
|  |  |  | YLD | YLL | Total DALYs | DALYs per 1,000 cases | DHC  (million €) | Patient costs (million €) | Productivity losses (million €) | Total costs (million €) | Costs per 1,000 cases  (*1,000 €) |
| **Overall** | |  | 84  (32-160) | 17  (0-65) | 102  (39-190) | 3  (2-5) | 1  (0.7-1.5) | 0.2  (0.1-0.3) | 12.5  (4.2-24.5) | 13.7  (5-26.1) | 380  (333-470) |
| **Per million inhabitants** | |  | 5  (2-9) | 1  (0-4) | 6  (2-11) | - | 0.06  (0.04-0.09) | 0.01  (0.01-0.02) | 0.7  (0.2-1.4) | 0.8  (0.3-1.5) | - |
| **Acute episode** | |  | 75  (29-142) | 17  (0-65) | 92  (36-173) | 2.7  (1.7-5.1) | 1  (0.7-1.4) | 0.1  (0.1-0.3) | 11.4  (3.9-22.2) | 12.5  (4.7-23.7) | 348  (309-433) |
| **Long-term manifestations** | **Diarrhea** | | 4  (1-11) | 0  (0-0) | 4  (1-11) | 0.1  (0.03-0.24) | 0  (0-0) | 0.01  (0-0.03) | 0.75  (0.13-2.31) | 0.8  (0.1-2.3) | 21  (6-50) |
|  | **Joint pain** | | 6  (2-13) | 0  (0-0) | 6  (2-13) | 0.15  (0.09-0.22) | 0.02  (0.01-0.06) | 0  (0-0.01) | 0.37  (0.1-0.83) | 0.4  (0.1-0.9) | 11  (6-15) |
|  | **Total** | | 9  (3-22) | 0  (0-0) | 9  (3-22) | 0.3  (0.1-0.4) | 0.02  (0.01-0.06) | 0.02  (0.01-0.03) | 1.12  (0.27-2.92) | 1.2  (0.3-3) | 31.5  (15.3-61.2) |
| **By species** | ***C. hominis*** | | 11  (4-20) | 2  (0-15) | 12  (4-28) | 2.8  (1.9-6.6) | 0.1  (0.1-0.2) | 0.02  (0.01-0.04) | 1.6  (0.5-3.1) | 1.7  (0.6-3.3) | 376  (331-462) |
|  | ***C. parvum*** | | 74  (28-140) | 16  (0-56) | 89  (35-166) | 3  (2-5.4) | 0.9  (0.6-1.3) | 0.14  (0.05-0.26) | 10.9  (3.7-21.4) | 12  (4.4-22.8) | 380  (333-471) |

*The DALYs are discounted at 1.5% per year according to the Dutch guidelines for health economics(38); ^#^costs are not discounted since all costs happened within the year of infection.

**Year: 2015**

|  | | | DALYs* | | | | Costs^#^ | | | | |
| --- | --- | --- | --- | --- | --- | --- | --- | --- | --- | --- | --- |
|  |  |  | YLD | YLL | Total DALYs | DALYs per 1,000 cases | DHC  (million €) | Patient costs (million €) | Productivity losses (million €) | Total costs (million €) | Costs per 1,000 cases  (*1,000 €) |
| **Overall** | |  | 152  (58-287) | 31  (0-108) | 183  (72-337) | 3  (2-5) | 1.8  (1.2-2.6) | 0.3  (0.1-0.5) | 24  (8.6-46) | 26.1  (10.1-48.9) | 407  (346-534) |
| **Per million inhabitants** | |  | 9  (3-17) | 2  (0-6) | 11  (4-20) | - | 0.11  (0.07-0.15) | 0.02  (0.01-0.03) | 1.4  (0.5-2.7) | 1.5  (0.6-2.9) | - |
| **Acute episode** | |  | 135  (52-254) | 31  (0-108) | 166  (66-305) | 2.7  (1.7-5) | 1.8  (1.2-2.5) | 0.3  (0.1-0.5) | 22  (8-41.9) | 24  (9.4-44.6) | 376  (321-497) |
| **Long-term manifestations** | **Diarrhea** | | 7  (1-20) | 0  (0-0) | 7  (1-20) | 0.1  (0.03-0.24) | 0  (0-0) | 0.02  (0.01-0.05) | 1.36  (0.23-4.15) | 1.4  (0.2-4.2) | 21  (6-50) |
|  | **Joint pain** | | 10  (3-23) | 0  (0-0) | 10  (3-23) | 0.15  (0.09-0.22) | 0.04  (0.01-0.1) | 0.01  (0-0.02) | 0.66  (0.18-1.48) | 0.7  (0.2-1.6) | 11  (6-15) |
|  | **Total** | | 17  (5-39) | 0  (0-0) | 17  (5-39) | 0.3  (0.1-0.4) | 0.04  (0.01-0.1) | 0.03  (0.01-0.06) | 2.02  (0.48-5.26) | 2.1  (0.5-5.4) | 31.6  (15.3-61.2) |
| **By species** | ***C. hominis*** | | 73  (28-139) | 13  (0-56) | 87  (33-163) | 2.9  (1.9-5.3) | 0.9  (0.6-1.3) | 0.14  (0.05-0.26) | 10.7  (3.6-21.1) | 11.8  (4.3-22.5) | 376  (331-462) |
|  | ***C. parvum*** | | 78  (30-149) | 18  (0-62) | 96  (38-178) | 3  (2-5.5) | 1  (0.6-1.4) | 0.15  (0.06-0.28) | 13.3  (5-24.9) | 14.4  (5.8-26.5) | 436  (358-605) |

*The DALYs are discounted at 1.5% per year according to the Dutch guidelines for health economics(38); ^#^costs are not discounted since all costs happened within the year of infection.

**Year: 2016**

|  | | | DALYs* | | | | Costs^#^ | | | | |
| --- | --- | --- | --- | --- | --- | --- | --- | --- | --- | --- | --- |
|  |  |  | YLD | YLL | Total DALYs | DALYs per 1,000 cases | DHC  (million €) | Patient costs (million €) | Productivity losses (million €) | Total costs (million €) | Costs per 1,000 cases  (*1,000 €) |
| **Overall** | |  | 181  (69-343) | 37  (0-126) | 218  (87-401) | 3  (2-5) | 2.2  (1.5-3.1) | 0.3  (0.1-0.6) | 28.5  (10.2-54.6) | 31  (12-58.1) | 406  (345-530) |
| **Per million inhabitants** | |  | 11  (4-20) | 2  (0-7) | 13  (5-24) | - | 0.13  (0.09-0.18) | 0.02  (0.01-0.04) | 1.7  (0.6-3.2) | 1.8  (0.7-3.4) | - |
| **Acute episode** | |  | 161  (62-303) | 37  (0-126) | 198  (79-364) | 2.7  (1.7-4.9) | 2.1  (1.4-3) | 0.3  (0.1-0.6) | 26.1  (9.5-49.7) | 28.5  (11.2-53) | 374  (320-493) |
| **Long-term manifestations** | **Diarrhea** | | 8  (1-24) | 0  (0-0) | 8  (1-24) | 0.1  (0.03-0.24) | 0  (0-0) | 0.03  (0.01-0.05) | 1.61  (0.27-4.95) | 1.6  (0.3-5) | 21  (6-50) |
|  | **Joint pain** | | 12  (3-27) | 0  (0-0) | 12  (3-27) | 0.15  (0.09-0.22) | 0.05  (0.01-0.12) | 0.01  (0-0.02) | 0.79  (0.21-1.77) | 0.8  (0.2-1.9) | 11  (6-15) |
|  | **Total** | | 20  (5-46) | 0  (0-0) | 20  (5-46) | 0.3  (0.1-0.4) | 0.05  (0.01-0.12) | 0.03  (0.01-0.07) | 2.4  (0.57-6.26) | 2.5  (0.6-6.4) | 31.5  (15.3-61.2) |
| **By species** | ***C. hominis*** | | 133  (51-252) | 27  (0-96) | 160  (63-295) | 2.9  (2-5.2) | 1.6  (1.1-2.3) | 0.25  (0.09-0.47) | 19.5  (6.5-38.3) | 21.4  (7.8-40.9) | 376  (331-462) |
|  | ***C. parvum*** | | 48  (18-90) | 10  (0-41) | 58  (22-109) | 3  (2-5.6) | 0.6  (0.4-0.8) | 0.09  (0.03-0.17) | 9  (3.7-16.4) | 9.6  (4.1-17.4) | 489  (379-739) |

*The DALYs are discounted at 1.5% per year according to the Dutch guidelines for health economics(38); ^#^costs are not discounted since all costs happened within the year of infection.

**Year: 2017**

|  | | | DALYs* | | | | Costs^#^ | | | | |
| --- | --- | --- | --- | --- | --- | --- | --- | --- | --- | --- | --- |
|  |  |  | YLD | YLL | Total DALYs | DALYs per 1,000 cases | DHC  (million €) | Patient costs (million €) | Productivity losses (million €) | Total costs (million €) | Costs per 1,000 cases  (*1,000 €) |
| **Overall** | |  | 114  (43-216) | 23  (0-83) | 137  (54-255) | 3  (2-5) | 1.4  (0.9-2) | 0.2  (0.1-0.4) | 17.6  (6.1-34) | 19.2  (7.2-36.2) | 396  (341-506) |
| **Per million inhabitants** | |  | 7  (3-13) | 1  (0-5) | 8  (3-15) | - | 0.08  (0.05-0.12) | 0.01  (0.01-0.02) | 1  (0.4-2) | 1.1  (0.4-2.1) | - |
| **Acute episode** | |  | 101  (39-191) | 23  (0-83) | 125  (49-231) | 2.7  (1.7-5.1) | 1.3  (0.9-1.9) | 0.2  (0.1-0.4) | 16  (5.7-30.9) | 17.6  (6.7-32.9) | 364  (316-469) |
| **Long-term manifestations** | **Diarrhea** | | 5  (1-15) | 0  (0-0) | 5  (1-15) | 0.1  (0.03-0.24) | 0  (0-0) | 0.02  (0.01-0.03) | 1.02  (0.17-3.13) | 1  (0.2-3.2) | 21  (6-50) |
|  | **Joint pain** | | 8  (2-17) | 0  (0-0) | 8  (2-17) | 0.15  (0.09-0.22) | 0.03  (0.01-0.08) | 0.01  (0-0.01) | 0.5  (0.13-1.12) | 0.5  (0.1-1.2) | 11  (6-15) |
|  | **Total** | | 13  (3-29) | 0  (0-0) | 13  (3-29) | 0.3  (0.1-0.4) | 0.03  (0.01-0.08) | 0.02  (0.01-0.04) | 1.52  (0.36-3.96) | 1.6  (0.4-4.1) | 31.6  (15.4-61.4) |
| **By species** | ***C. hominis*** | | 36  (14-69) | 7  (0-33) | 44  (16-84) | 2.9  (1.9-5.8) | 0.4  (0.3-0.6) | 0.07  (0.03-0.13) | 5.3  (1.8-10.5) | 5.8  (2.1-11.1) | 377  (332-463) |
|  | ***C. parvum*** | | 78  (30-148) | 16  (0-60) | 94  (37-174) | 2.9  (2-5.3) | 0.9  (0.6-1.3) | 0.15  (0.05-0.28) | 12.2  (4.4-23.5) | 13.3  (5.1-25) | 404  (344-526) |

*The DALYs are discounted at 1.5% per year according to the Dutch guidelines for health economics(38); ^#^costs are not discounted since all costs happened within the year of infection.

**Table S7. Results of the scenarios and sensitivity analyses performed. Point estimates (95% uncertainty interval) for disability-adjusted life years (DALY) and costs of illness (COI). Estimations performed for year 2017.**

|  | **Scenario & sensitivity analyses** | **DALY** | | | | **Costs** | | | | |
| --- | --- | --- | --- | --- | --- | --- | --- | --- | --- | --- |
|  |  | **Total DALY** | **YLL** | **YLD** | **Per 1,000 cases** | **Total COI** | **DHC** | **Patient costs** | **Productivity losses** | **Per 1,000 cases** |
| **1** | **BASE CASE** | **137 (55-257)** | **23 (0-82)** | **114 (44-218)** | **2.9 (2-5.4)** | **19 (7-37)** | **1.4 (0.9-2)** | **0.2 (0.1-0.4)** | **18 (6-34)** | **0.4 (0.3-0.5)** |
| 2 | Effects elapsing for more than 1 year are not discounted | 140 (55-266) | 26 (0-98) | 114 (44-218) | 3.0 (2.0-5.8) | 19 (7-37) | 1.4 (0.9-2) | 0.2 (0.1-0.4) | 18 (6-34) | 0.4 (0.3-0.5) |
| 3 | For years of life lost, Dutch life-expectancy is used | 135 (55-252) | 21 (0-75) | 114 (44-218) | 2.9 (2.0-5.2) | 19 (7-37) | 1.4 (0.9-2) | 0.2 (0.1-0.4) | 18 (6-34) | 0.4 (0.3-0.5) |
| 4 | Age-distribution of reported cases does not vary between species | 137 (55-257) | 23 (0-82) | 114 (44-218) | 2.9 (2.0-5.4) | 19 (7-37) | 1.4 (0.9-2) | 0.2 (0.1-0.4) | 18 (6-35) | 0.4 (0.3-0.5) |
| 5 | Proportion of all reported cases that are C. hominis is 52% for all years | 137 (55-257) | 23 (0-82) | 114 (44-218) | 2.9 (2-5.4) | 19 (7-37) | 1.4 (0.9-2) | 0.2 (0.1-0.4) | 18 (6-34) | 0.4 (0.3-0.5) |
| 6 | Proportion of all reported cases that are C. hominis is 13% for all years | 137 (55-256) | 23 (0-84) | 114 (43-217) | 2.9 (2.0-5.4) | 19 (7-36) | 1.4 (0.9-2) | 0.2 (0.1-0.4) | 17 (6-34) | 0.4 (0.3-0.5) |
| 7 | Proportion of all reported cases that are C. hominis is 48% for all years | 137 (55-259) | 23 (0-82) | 114 (44-218) | 2.9 (2.0-5.4) | 19 (7-37) | 1.4 (0.9-2) | 0.2 (0.1-0.4) | 18 (6-35) | 0.4 (0.3-0.5) |
| 8 | MF for underascertainment of symptomatic cases: no age variation | 137 (55-257) | 23 (0-82) | 114 (44-218) | 2.9 (2.0-5.4) | 15 (6-27) | 1.4 (0.9-2) | 0.2 (0.1-0.4) | 13 (5-25) | 0.3 (0.3-0.4) |
| 9 | MF for estimation of cases in the GP: no age variation | 137 (55-257) | 23 (0-82) | 114 (44-218) | 2.9 (2.0-5.4) | 19 (7-36) | 1.4 (0.9-2) | 0.2 (0.1-0.4) | 17 (6-34) | 0.4 (0.3-0.5) |
| 10 | MF for underreporting of hospitalized and deceased: no age variation | 137 (55-257) | 23 (0-82) | 114 (44-218) | 2.9 (2.0-5.4) | 19 (7-36) | 1.4 (0.9-2) | 0.2 (0.1-0.4) | 17 (6-34) | 0.4 (0.3-0.5) |
| 11 | Scenarios 6 + 7 + 8 | 137 (55-257) | 23 (0-82) | 114 (44-218) | 2.9 (2.0-5.4) | 14 (5-26) | 1.4 (0.9-2) | 0.2 (0.1-0.4) | 12 (4-23) | 0.3 (0.2-0.4) |
| 12 | Lower MF for underascertainment of symptomatic cases | 105 (43-196) | 23 (0-82) | 82 (34-151) | 3.9 (2.4-8.1) | 13 (5-24) | 1.4 (0.9-1.9) | 0.1 (0.1-0.3) | 11 (4-22) | 0.5 (0.4-0.6) |
| 13 | Lower MF for estimation of cases presenting to the GP | 120 (45-233) | 23 (0-82) | 97 (35-192) | 2.6 (1.7-4.8) | 18 (7-35) | 1.2 (0.8-1.6) | 0.2 (0.1-0.4) | 17 (6-33) | 0.4 (0.3-0.5) |
| 14 | Scenarios 11 + 12 | 88 (33-172) | 23 (0-82) | 65 (25-124) | 3.3 (2.0-7.1) | 12 (5-22) | 1.1 (0.8-1.6) | 0.1 (0-0.2) | 11 (4-20) | 0.4 (0.3-0.6) |
| 15 | There is no mortality due to *Cryptosporidium* | 114 (44-218) | 0 (0-0) | 114 (44-218) | 2.4 (1.8-3.2) | 19 (7-37) | 1.4 (0.9-2) | 0.2 (0.1-0.4) | 18 (6-34) | 0.4 (0.3-0.5) |
| 16 | Age-distribution of deceased cases similar to the one in TESSy | 191 (62-399) | 77 (0-256) | 114 (44-218) | 4.3 (2-11.2) | 19 (7-37) | 1.4 (0.9-2) | 0.2 (0.1-0.4) | 18 (6-34) | 0.4 (0.3-0.5) |
| 17 | Incidence of recurrent diarrhea in mild cases similar to moderate and severe | 140 (56-264) | 23 (0-82) | 117 (44-225) | 3.0 (2.0-5.5) | 20 (7-38) | 1.4 (0.9-2) | 0.2 (0.1-0.4) | 18 (6-36) | 0.4 (0.3-0.5) |
| 18 | Incidence of recurrent diarrhea similar in children and in adults | 137 (55-257) | 23 (0-82) | 114 (44-218) | 2.9 (2.0-5.4) | 19 (7-37) | 1.4 (0.9-2) | 0.2 (0.1-0.4) | 18 (6-34) | 0.4 (0.3-0.5) |
| 19 | Incidence of recurrent gastrointestinal symptoms (overall) computed | 148 (58-280) | 23 (0-82) | 125 (46-242) | 3.1 (2.1-5.6) | 21 (8-42) | 1.4 (0.9-2) | 0.3 (0.1-0.5) | 20 (7-39) | 0.4 (0.4-0.6) |
| 20 | Varying severity of recurrent diarrhea episodes | 143 (57-269) | 23 (0-82) | 120 (46-230) | 3.1 (2.1-5.6) | 19 (7-37) | 1.6 (1.1-2.2) | 0.2 (0.1-0.4) | 18 (6-34) | 0.4 (0.3-0.5) |
| 21 | **16** + Costs of mild recurrent diarrhea is similar to moderate diarrhea cases | 143 (57-269) | 23 (0-82) | 120 (46-230) | 3.1 (2.1-5.6) | 20 (8-38) | 1.9 (1.2-2.9) | 0.2 (0.1-0.4) | 18 (6-34) | 0.4 (0.4-0.5) |
| 22 | Double duration and number of recurrent diarrhea episodes | 152 (60-289) | 23 (0-82) | 129 (48-254) | 3.2 (2.2-5.8) | 22 (8-44) | 1.4 (0.9-2) | 0.2 (0.1-0.4) | 21 (7-42) | 0.5 (0.4-0.6) |
| 23 | Scenarios 16 + 17 + 19 + 20 + 21 | 204 (74-428) | 23 (0-82) | 180 (59-395) | 4.3 (2.6-7.3) | 27 (9-54) | 3.2 (1.7-5.3) | 0.3 (0.1-0.6) | 23 (7-49) | 0.5 (0.4-0.7) |
| 24 | Mild cases of *Cryptosporidium* cannot result in joint pain | 131 (53-245) | 23 (0-82) | 107 (42-204) | 2.8 (1.8-5.3) | 19 (7-36) | 1.4 (0.9-1.9) | 0.2 (0.1-0.4) | 17 (6-34) | 0.4 (0.3-0.5) |
| 25 | Children <15 years cannot experience joint pain | 135 (54-254) | 23 (0-82) | 112 (43-215) | 2.9 (1.9-5.4) | 19 (7-36) | 1.4 (0.9-2) | 0.2 (0.1-0.4) | 17 (6-34) | 0.4 (0.3-0.5) |
| 26 | Infections by *C. parvum* cannot result in joint pain | 132 (53-247) | 23 (0-82) | 109 (42-207) | 2.8 (1.9-5.3) | 19 (7-36) | 1.4 (0.9-1.9) | 0.2 (0.1-0.4) | 17 (6-34) | 0.4 (0.3-0.5) |
| 27 | Higher duration for joint pain is assumed | 172 (67-327) | 23 (0-82) | 149 (54-291) | 3.6 (2.6-6.1) | 19 (7-37) | 1.4 (0.9-2) | 0.2 (0.1-0.4) | 18 (6-34) | 0.4 (0.3-0.5) |
| 28 | Scenarios 24 + 25 + 26 + 27 | 131 (53-245) | 23 (0-82) | 108 (42-205) | 2.8 (1.8-5.3) | 19 (7-35) | 1.3 (0.9-1.9) | 0.2 (0.1-0.4) | 17 (6-33) | 0.4 (0.3-0.5) |
| 29 | Dizziness and fatigue are included as a long-term manifestations | 138 (55-259) | 23 (0-82) | 115 (44-221) | 3.0 (2.0-5.4) | 19 (7-37) | 1.4 (0.9-2) | 0.2 (0.1-0.4) | 18 (6-34) | 0.4 (0.3-0.5) |
| 30 | All scenarios that are more conservative | 61 (23-115) | 0 (0-0) | 61 (23-115) | 2.1 (1.6-2.9) | 8 (3-15) | 1.1 (0.8-1.6) | 0.1 (0-0.2) | 7 (2-13) | 0.3 (0.2-0.4) |
| 31 | All scenarios that are less conservative | 395 (121-846) | 113 (0-389) | 282 (86-647) | 8.6 (4.1-19.3) | 34 (11-74) | 4.7 (2.4-8.1) | 0.4 (0.1-0.8) | 29 (9-65) | 0.7 (0.5-1) |

DALY: Disability-Adjusted Life Years; YLL: Years of Life Lost; YLD: Years lived with disability; COI: cost of illness; DHC: Direct healthcare costs; MF: Multiplication Factors

**References**

1. Abal-Fabeiro JL, Maside X, Llovo J, Bartolome C. Aetiology and epidemiology of human cryptosporidiosis cases in Galicia (NW Spain), 2000-2008. Epidemiology and infection. 2015;143(14):3022-35.

2. Adler S, Widerstrom M, Lindh J, Lilja M. Symptoms and risk factors of Cryptosporidium hominis infection in children: data from a large waterborne outbreak in Sweden. Parasitology research. 2017;116(10):2613-8.

3. Widerstrom M, Schonning C, Lilja M, Lebbad M, Ljung T, Allestam G, et al. Large outbreak of Cryptosporidium hominis infection transmitted through the public water supply, Sweden. Emerging infectious diseases. 2014;20(4):581-9.

4. Bouzid M, Hunter PR, Chalmers RM, Tyler KM. Cryptosporidium pathogenicity and virulence. Clinical microbiology reviews. 2013;26(1):115-34.

5. Cassini A, Colzani E, Pini A, Mangen MJ, Plass D, McDonald SA, et al. Impact of infectious diseases on population health using incidence-based disability-adjusted life years (DALYs): results from the Burden of Communicable Diseases in Europe study, European Union and European Economic Area countries, 2009 to 2013. Euro surveillance : bulletin Europeen sur les maladies transmissibles = European communicable disease bulletin. 2018;23(16).

6. Corso PS, Kramer MH, Blair KA, Addiss DG, Davis JP, Haddix AC. Cost of illness in the 1993 waterborne Cryptosporidium outbreak, Milwaukee, Wisconsin. Emerging infectious diseases. 2003;9(4):426-31.

7. MacKenzie WR, Schell WL, Blair KA, Addiss DG, Peterson DE, Hoxie NJ, et al. Massive outbreak of waterborne cryptosporidium infection in Milwaukee, Wisconsin: recurrence of illness and risk of secondary transmission. Clin Infect Dis. 1995;21(1):57-62.

8. Dietz V, Vugia D, Nelson R, Wicklund J, Nadle J, McCombs KG, et al. Active, multisite, laboratory-based surveillance for Cryptosporidium parvum. The American journal of tropical medicine and hygiene. 2000;62(3):368-72.

9. Ethelberg S, Lisby M, Vestergaard LS, Enemark HL, Olsen KE, Stensvold CR, et al. A foodborne outbreak of Cryptosporidium hominis infection. Epidemiology and infection. 2009;137(3):348-56.

10. Haagsma JA, Siersema PD, De Wit NJ, Havelaar AH. Disease burden of post-infectious irritable bowel syndrome in The Netherlands. Epidemiology and infection. 2010;138(11):1650-6.

11. Hoxie NJ, Davis JP, Vergeront JM, Nashold RD, Blair KA. Cryptosporidiosis-associated mortality following a massive waterborne outbreak in Milwaukee, Wisconsin. American journal of public health. 1997;87(12):2032-5.

12. Hunter PR, Hughes S, Woodhouse S, Syed Q, Verlander NQ, Chalmers RM, et al. Sporadic cryptosporidiosis case-control study with genotyping. Emerging infectious diseases. 2004;10(7):1241-9.

13. Hunter PR, Hughes S, Woodhouse S, Raj N, Syed Q, Chalmers RM, et al. Health sequelae of human cryptosporidiosis in immunocompetent patients. Clin Infect Dis. 2004;39(4):504-10.

14. Igloi Z, Mughini-Gras L, Nic Lochlainn L, Barrasa A, Sane J, Mooij S, et al. Long-term sequelae of sporadic cryptosporidiosis: a follow-up study. European journal of clinical microbiology & infectious diseases : official publication of the European Society of Clinical Microbiology. 2018.

15. Nic Lochlainn L, Sane J, Schimmer B, Mooij S, Roelfsema J, Kortbeek T, et al. Risk factors for sporadic cryptosporidiosis in the Netherlands: Analysis of a three-year population based case control study coupled with genotyping, 2013-2016. . In press. 2018.

16. Insulander M, de Jong B, Svenungsson B. A food-borne outbreak of cryptosporidiosis among guests and staff at a hotel restaurant in Stockholm county, Sweden, September 2008. Euro surveillance : bulletin Europeen sur les maladies transmissibles = European communicable disease bulletin. 2008;13(51).

17. Insulander M, Silverlas C, Lebbad M, Karlsson L, Mattsson JG, Svenungsson B. Molecular epidemiology and clinical manifestations of human cryptosporidiosis in Sweden. Epidemiology and infection. 2013;141(5):1009-20.

18. Mead PS, Slutsker L, Dietz V, McCaig LF, Bresee JS, Shapiro C, et al. Food-related illness and death in the United States. Emerging infectious diseases. 1999;5(5):607-25.

19. McCann R, Jones R, Snow J, Cleary P, Burgess S, Bothra V, et al. An outbreak of cryptosporidiosis at a swimming club--can rapid field epidemiology limit the spread of illness? Epidemiology and infection. 2014;142(1):51-5.

20. Rehn M, Wallensten A, Widerstrom M, Lilja M, Grunewald M, Stenmark S, et al. Post-infection symptoms following two large waterborne outbreaks of Cryptosporidium hominis in Northern Sweden, 2010-2011. BMC public health. 2015;15:529.

21. Schierenberg A, Broekhuizen BDL, Nipshagen MD, Kommer MBJ, Bruijning-Verhagen PCJ, van Delft S, et al. Guideline adherence for diagnostic faeces testing in primary care patients with gastroenteritis. Fam Pract. 2017;34(6):692-6.

22. Stiff RE, Davies AP, Mason BW, Hutchings HA, Chalmers RM. Long-term health effects after resolution of acute Cryptosporidium parvum infection: a 1-year follow-up of outbreak-associated cases. Journal of medical microbiology. 2017;66(11):1607-11.

23. Tam CC, Rodrigues LC, Viviani L, Dodds JP, Evans MR, Hunter PR, et al. Longitudinal study of infectious intestinal disease in the UK (IID2 study): incidence in the community and presenting to general practice. Gut. 2012;61(1):69-77.

24. Laboratory-based surveillance for Cryptosporidium in France, 2006-2009. Euro surveillance : bulletin Europeen sur les maladies transmissibles = European communicable disease bulletin. 2010;15(33):19642.

25. Widerstrom M, Omberg M, Svensson I, Rundvik M, Wistrom J, editors. Characteristics of patients hospitalized with Cryptosporidium hominis infection during a large outbreak of waterborne cryptosporidiosis in Östersund, Sweden. ECAIDE; 2011 6-8 November; Stockholm.

26. de Wit MA, Kortbeek LM, Koopmans MP, de Jager CJ, Wannet WJ, Bartelds AI, et al. A comparison of gastroenteritis in a general practice-based study and a community-based study. Epidemiology and infection. 2001;127(3):389-97.

27. Chalmers RM, Davies AP. Minireview: clinical cryptosporidiosis. Experimental parasitology. 2010;124(1):138-46.

28. Arslan H, Inci EK, Azap OK, Karakayali H, Torgay A, Haberal M. Etiologic agents of diarrhea in solid organ recipients. Transplant infectious disease : an official journal of the Transplantation Society. 2007;9(4):270-5.

29. Frisby HR, Addiss DG, Reiser WJ, Hancock B, Vergeront JM, Hoxie NJ, et al. Clinical and epidemiologic features of a massive waterborne outbreak of cryptosporidiosis in persons with HIV infection. Journal of acquired immune deficiency syndromes and human retrovirology : official publication of the International Retrovirology Association. 1997;16(5):367-73.

30. Bandin F, Kwon T, Linas MD, Guigonis V, Valentin A, Cassaing S, et al. Cryptosporidiosis in paediatric renal transplantation. Pediatric nephrology (Berlin, Germany). 2009;24(11):2245-55.

31. Bachur TP, Vale JM, Coelho IC, Queiroz TR, Chaves Cde S. Enteric parasitic infections in HIV/AIDS patients before and after the highly active antiretroviral therapy. The Brazilian journal of infectious diseases : an official publication of the Brazilian Society of Infectious Diseases. 2008;12(2):115-22.

32. Ryom L, Boesecke C, Gisler V, Manzardo C, Rockstroh JK, Puoti M, et al. Essentials from the 2015 European AIDS Clinical Society (EACS) guidelines for the treatment of adult HIV-positive persons. HIV medicine. 2016;17(2):83-8.

33. Murray CJ, Vos T, Lozano R, Naghavi M, Flaxman AD, Michaud C, et al. Disability-adjusted life years (DALYs) for 291 diseases and injuries in 21 regions, 1990-2010: a systematic analysis for the Global Burden of Disease Study 2010. Lancet. 2012;380(9859):2197-223.

34. Statistics Netherlands (Centraal Bureau voor de Statistiek). [Deceased people; main causes of death (short list), age, gender], in Dutch. Available at: <http://statline.cbs.nl/>. Accessed 13 August 2018.

35. Statistics Netherlands (Centraal Bureau voor de Statistiek). [Life expectancy; sex, age (per year and period of five years)], in Dutch. Available at: <http://statline.cbs.nl/>. Accessed 18 May 2018.

36. Hannu T, Mattila L, Rautelin H, Pelkonen P, Lahdenne P, Siitonen A, et al. Campylobacter-triggered reactive arthritis: a population-based study. Rheumatology (Oxford). 2002;41(3):312-8.

37. Locht H, Krogfelt KA. Comparison of rheumatological and gastrointestinal symptoms after infection with Campylobacter jejuni/coli and enterotoxigenic Escherichia coli. Annals of the rheumatic diseases. 2002;61(5):448-52.

38. Hakkaart-van Roijen L, van der Linden N, Bouwmans C, Kanters T, Swan Tan S. [Costs guide: Methodology of costs research and reference prices for economic evaluations in health care](Dutch). Diemen (The Netherlands): Zorginstituut Nederland (ZIN); 2016.

39. Haagsma JA, Maertens de Noordhout C, Polinder S, Vos T, Havelaar AH, Cassini A, et al. Assessing disability weights based on the responses of 30,660 people from four European countries. Popul Health Metr. 2015;13:10.

40. Mangen MJ, Bouwknegt M, Friesema IH, Haagsma JA, Kortbeek LM, Tariq L, et al. Cost-of-illness and disease burden of food-related pathogens in the Netherlands, 2011. International journal of food microbiology. 2015;196:84-93.

41. Doorduyn Y, Van Pelt W, Havelaar AH. The burden of infectious intestinal disease (IID) in the community: a survey of self-reported IID in The Netherlands. Epidemiology and infection. 2012;140(7):1185-92.

42. Donker G. NIVEL Primary Care Database – Sentinel Practices 2017. The Netherlands year report. Utrecht; 2018.

43. Friesema IH, Lugner AK, van Duynhoven YT, Group GEW. Costs of gastroenteritis in the Netherlands, with special attention for severe cases. European journal of clinical microbiology & infectious diseases : official publication of the European Society of Clinical Microbiology. 2012;31(8):1895-900.

44. Mangen MJ, Friesema IH, Haagsma JA, van Pelt W. Disease burden of food-related pathogens in the Netherlands, 2016. Bilthoven (the Netherlands): RIVM; 2017.

45. van den Brandhof WE, De Wit GA, de Wit MA, van Duynhoven YT. Costs of gastroenteritis in The Netherlands. Epidemiology and infection. 2004;132(2):211-21.

46. de Wit MA, Koopmans MP, Kortbeek LM, van Leeuwen NJ, Vinje J, van Duynhoven YT. Etiology of gastroenteritis in sentinel general practices in the netherlands. Clin Infect Dis. 2001;33(3):280-8.
